# Single-cell RNA sequencing reveals enhanced antitumor immunity after combined application of PD-1 inhibitor and Shenmai injection in non-small cell lung cancer

**DOI:** 10.1186/s12964-023-01184-3

**Published:** 2023-07-10

**Authors:** Dingyi Yu, Penghui Yang, Xiaoyan Lu, Shaoze Huang, Li Liu, Xiaohui Fan

**Affiliations:** 1 Pharmaceutical Informatics Institute, College of Pharmaceutical Sciences, Zhejiang University, Hangzhou, China; 2 National Key Laboratory of Chinese Medicine Modernization, Innovation Center of Yangtze River Delta, Zhejiang University, Jiaxing, China; 3 Innovation Center in Zhejiang University, State Key Laboratory of Component-Based Chinese Medicine, Hangzhou, China; 4 Westlake Laboratory of Life Sciences and Biomedicine, Hangzhou, China; 5 Department of Infectious Diseases, The Second Affiliated Hospital, Zhejiang University School of Medicine, Hangzhou, China; 6 Zhejiang Engineering Research Center for Advanced Manufacturing of Traditional Chinese Medicine, Huzhou, China

**Keywords:** Non-small cell lung cancer, Single-cell RNA sequencing, Programmed death-1 inhibitor, Shenmai injection, Natural killer cells

## Abstract

**Background:**

Immune checkpoint inhibitors (ICIs) have altered the clinical management of non-small cell lung cancer (NSCLC). However, the low response rate, severe immune-related adverse events (irAEs), and hyperprogressive disease following ICIs monotherapy require attention. Combination therapy may overcome these limitations and traditional Chinese medicine with immunomodulatory effects provides a promising approach. Shenmai injection (SMI) is a clinically effective adjuvant treatment for cancer with chemotherapy and radiotherapy. Therefore, the combined effects and mechanisms of SMI and programmed death-1 (PD-1) inhibitor against NSCLC was focused on this study.

**Methods:**

A Lewis lung carcinoma mouse model and a lung squamous cell carcinoma humanized mouse model were used to investigate the combined efficacy and safety of SMI and PD-1 inhibitor. The synergistic mechanisms of the combination therapy against NSCLC were explored using single-cell RNA sequencing. Validation experiments were performed using immunofluorescence analysis, in vitro experiment, and bulk transcriptomic datasets.

**Results:**

In both models, combination therapy alleviated tumor growth and prolonged survival without increasing irAEs. The *GZMA*^high^ and *XCL1*^high^ natural killer (NK) cell subclusters with cytotoxic and chemokine signatures increased in the combination therapy, while malignant cells from combination therapy were mainly in the apoptotic state, suggesting that mediating tumor cell apoptosis through NK cells is the main synergistic mechanisms of combination therapy. In vitro experiment confirmed that combination therapy increased secretion of Granzyme A by NK cells. Moreover, we discovered that PD-1 inhibitor and SMI combination blocked inhibitory receptors on NK and T cells and restores their antitumoral activity in NSCLC better than PD-1 inhibitor monotherapy, and immune and stromal cells exhibited a decrease of angiogenic features and attenuated cancer metabolism reprogramming in microenvironment of combination therapy.

**Conclusions:**

This study demonstrated that SMI reprograms tumor immune microenvironment mainly by inducing NK cells infiltration and synergizes with PD-1 inhibitor against NSCLC, suggested that targeting NK cells may be an important strategy for combining with ICIs.

Video Abstract

**Supplementary Information:**

The online version contains supplementary material available at 10.1186/s12964-023-01184-3.

## Background

Lung cancer is the second most diagnosed cancer (11.4%) and primary cause of cancer death (18%) in the world according to the 2020 Global Cancer Statistics [1]. In China, lung cancer is the leading cause of cancer-related morbidity and mortality, with approximately 787,000 new diagnoses and 630,500 deaths per year [2]. Non-small cell lung cancer (NSCLC) is the predominant type of this disease and accounts for about 85% of all cases. NSCLC includes lung squamous cell carcinoma (LUSC), lung adenocarcinoma (LUAD), and large cell cancers. LUAD accounts for 40% of all lung cancer cases and benefits from targeted therapy, whereas LUSC accounts for 30% of NSCLC and the survival benefit from combined chemotherapy and targeted therapy is limited [3, 4]. Since the advent of immune checkpoint inhibitors (ICIs), the landscape of NSCLC treatment has strikingly changed. Programmed death-1/programmed death-ligand 1 (PD-1/PD-L1) inhibitors are ICIs that have been shown to significantly prolong the median progression-free survival (PFS) and overall survival (OS) in patients with NSCLC, including those with LUAD and LUSC [3]. The U.S. Food and Drug Administration has approved pembrolizumab (a PD-1 inhibitor) as a first-line therapy for tumors with a PD-L1 tumor proportion score ≥ 1% in NSCLC, cemiplimab (also a PD-1 inhibitor) for patients with PD-L1 expression ≥ 50%, and atezolizumab (a PD-L1 inhibitor) for patients with high PD-L1-expressing metastatic NSCLC [5]. However, the overall objective response rate for PD-1 and PD-L1 inhibitors in NSCLC is unsatisfactory (less than 20%) [6], and PD-1 resistance may appear in patients with advanced NSCLC [7]. Moreover, accelerated and unexpected progression in tumor volume, known as hyperprogressive disease (HPD), may occur with treatment with ICIs [8]. Importantly, numerous immune-related adverse events (irAEs) have been reported in patients with NSCLC treated with PD-1 or PD-L1 inhibitors, including pneumonitis, thyroiditis, and dermatitis [9].

Combination therapies such as PD-1/PD-L1 inhibitors combined with chemotherapy, targeted therapy, or other immunotherapies may be effective NSCLC treatments [10]. However, these combination therapies do not significantly improve the OS compared with that in pembrolizumab monotherapy in patients with PD-L1 ≥ 50%, and the risk of grade 3–5 irAEs is higher in combination therapy than in PD-L1 monotherapy [5]. Conversely, the incidence of acute kidney injury and nephritis is higher in pembrolizumab combination therapy than in placebo combination therapy, while the incidence of severe adverse events is higher in atezolizumab combination therapy than in chemotherapy [11, 12]. Therefore, there is an urgent clinical need to identify a combination therapy with strong antitumor effects and minimal irAEs.

Traditional Chinese medicine (TCM) contributes to adjuvant treatments of cancer, which not only reduce the side effects of chemotherapy and radiotherapy, but also increase antitumor efficacy. Shenmai injection (SMI), composed of red ginseng and *Radix Ophiopogonis*, is a popular Chinese patent medicine with immunomodulatory effects, including suppressing the release of inflammatory products from macrophages, increasing the activity of natural killer (NK) cells, and inhibiting differentiation of CD4^+^ T cells into regulatory T cells (Tregs) [13, 14]. SMI has been shown to exert synergistic and detoxifying effects in combination with antineoplastic agents (e.g., chemotherapy) for several cancer types, particularly lung cancer [15, 16]. For instance, SMI combined with chemotherapy increases the short-term treatment efficacy, markedly reduces the incidence of white blood cell, platelet, and hemoglobin decline, and alleviates liver injury in NSCLC compared with that in chemotherapy alone [15]. PD-1/PD-L1 inhibitors not only promote T cells but also influence other immune cells such as NK cells, dendritic cells (DCs), and tumor-associated macrophages to suppress tumor progression [17]. Given that both SMI and PD-1/PD-L1 inhibitors primarily induce immunomodulatory effects and impact similar immune cells, we investigated whether the combination of SMI and ICIs exerts synergistic antitumor effects with minimal irAEs in NSCLC.

Single-cell RNA sequencing (scRNA-seq) allows accurate analysis of the gene expression profiles of different types of immune, stromal, and tumor cells at the single-cell level. scRNA-seq also generates cell–cell interaction (CCI) networks in the tumor microenvironment (TME), which may aid in accurate identification of the mechanisms of action of antitumor drugs [18]. Therefore, scRNA-seq was used to explore the role and mechanism of PD-1/PD-L1 inhibitors in cancer treatment. Patil et al. [19] demonstrated that increased plasma cell signatures predict the OS of patients with NSCLC treated with atezolizumab using scRNA-seq analysis. Similarly, Zhang et al. [20] performed scRNA-seq on peripheral T cells from patients with NSCLC before and after PD-1 blockade and found that tumor-related CD4^+^ T cell clones exhibited higher cytotoxicity than controls.

Recently, humanized mouse models have been used to evaluate anti-PD-1-based therapies because of their several advantages [21]. First, human immune cells, including T, B, NK cells, macrophages, and DCs, are detected in the humanized mouse model. Second, humanized mice present interferon-gamma (IFN-γ) and antigen-specific cytotoxic T cell-producing responses [22]. Therefore, in this study, we evaluated the combined effects of SMI and PD-1 inhibitors in a Lewis lung carcinoma (LLC) C57BL/6 and LUSC NCI-H226 humanized mouse model and explored the synergistic mechanisms of combined therapy using scRNA-seq to provide insights on PD-1 inhibitor/SMI combination therapy for NSCLC.

## Methods

### Cell culture

Both LLC cells and LUSC NCI-H226 cells (Cell Bank of Shanghai Institutes for Biological Sciences, Chinese Academy of Sciences, Shanghai, China) were cultured in RPMI-1640 (Gibco, Grand Island, NY, USA) supplemented with 10% (v/v) heat-inactivated fetal bovine serum (Gibco, Grand Island, NY, USA), 100 U/mL penicillin, and 100 μg/mL streptomycin (Gibco, Grand Island, NY, USA) in a humidified atmosphere incubator at 37 °C with 95% air and 5% CO_2_. When cells reached 90% confluency, they were detached using 0.25% trypsin–EDTA (Gibco, Grand Island, NY, USA). The subcultivation ratio was 1:3.

### NSCLC mouse model

Male C57BL/6 mice (6–8 weeks old, 18–20 g) were purchased from Shanghai SLAC Laboratory Animal Co., Ltd. (Shanghai, China). Female NOD/ShiLtJGpt-*Prkdc*^*em26Cd52*^*Il2rg*^*em26Cd22*^/Gpt (NCG) mice were purchased from GemPharmatech Co., Ltd. (Nanjing, Jiangsu, China). Humanized NCG (HuNCG) mice were developed by intravenously injecting 1 × 10^5^ human CD34^+^ hematopoietic stem and progenitor cells into four-week-old female NCG mice as previously described [22]. Twelve weeks after transplantation, the engraftment level of human CD45^+^ cells in the peripheral blood of the mice was determined using flow cytometric quantification. Only NCG mice with over 25% human CD45^+^ cells were considered HuNCG mice [22]. Mice were housed under a 12-h light/dark cycle in a controlled environment with food and water provided ad libitum. Animal experiments were approved by the Animal Care and Use Committee of the Zhejiang University School of Medicine.

The LLC model was generated as follows: after three days of acclimation, LLC cells (5 × 10^5^ in 100 μL phosphate buffer saline [PBS]) were subcutaneously injected into the right flank of the C57BL/6 mice. Seven days after tumor inoculation, the mice were randomly divided into four groups: immunoglobulin G isotype control (IgG), PD-1 immune-checkpoint blockade antibody (PD-1), SMI monotherapy (SMI), and combination of anti-PD-1 and SMI (PD-1+SMI). Mice in the IgG and SMI groups were intraperitoneally (i.p.) injected with rat IgG2a isotype control (BP0089, Bio X Cell, Lebanon, NH, USA) at a dose of 10 mg/kg every three days, while mice in the PD-1 and PD-1+SMI groups were i.p. administered with 10 mg/kg anti-mouse PD-1 (CD279) RMPI-14 (BP0146, Bio X Cell, Lebanon, NH, USA) every three days. Moreover, mice in the IgG and PD-1 groups were i.p. administered with normal saline (batch number: 180108; Hangzhou Minsheng Pharmaceutical Group Co., Ltd., Hangzhou, Zhejiang, China) at 0.02 mL/g daily, and mice in the SMI and PD-1+SMI groups were i.p. injected with SMI (batch number: 2007152; Chiatai Qingchunbao Pharmaceutical Co., Ltd., Hangzhou, Zhejiang, China) at 0.02 mL/g (max equivalent dose of clinical use) daily. The SMI produced from Chiatai Qingchunbao Pharmaceutical Co., Ltd. meets the quality control indicators of national drug standards by the National Medical Products Administration (WS_3_-B-3428–98-2010). Specifically, the content of total ginsenosides in 1 mL of SMI calculated as the content of ginsenoside Re (C_48_H_82_O_18_) is 1.38 mg; the content of red ginseng in 1 mL of SMI calculated as the total content of ginsenoside Rg_1_ (C_42_H_72_O_14_) and ginsenoside Re (C_48_H_82_O_18_) combined is 0.24 mg; when calculated based on the content of ginsenoside Rb_1_ (C_54_H_92_O_23_), the content of red ginseng is 0.24 mg per 1 mL. Meanwhile, when calculated as the total content of ginsenoside Rg_1_ (C_42_H_72_O_14_), ginsenoside Re (C_48_H_82_O_18_), and ginsenoside Rb_1_ (C_54_H_92_O_23_), the content of red ginseng is 0.48 mg per 1 mL of SMI. In the survival experiment, drug administration was stopped after 28 days of treatment, and the survival period of the LLC tumor-bearing mice was observed and recorded until all the mice in the IgG group died. In the tumor volume measurement experiment, drug administration was stopped after 14 days of treatment, and tumor volumes were assessed every two days and calculated using the following equation: (length × width^2^)/2.

The NCI-H226 model was generated as follows: after three days of acclimation, NCI-H226 cells (2.5 × 10^6^ in 100 μL PBS medium) were subcutaneously injected into the right flank of HuNCG mice. After seven days of tumor inoculation, mice were randomly divided into the same four groups as mentioned in the LLC model. Mice in the IgG and SMI groups were i.p. administered with human IgG1 isotype control (BP0297, Bio X Cell, Lebanon, NH, USA) at a dose of 10 mg/kg twice a week, while mice in the PD-1 and PD-1+SMI groups were i.p. administered with Keytruda® Pembrolizumab anti-PD-1 inhibitor (S007468, Merck Sharp & Dohme Corp, New Jersey, USA) at a dose of 10 mg/kg twice a week. Meantime, mice in the IgG and PD-1 groups were i.p. administered with normal saline (batch number:180108; Hangzhou Minsheng Pharmaceutical Group Co., Ltd, Hangzhou, Zhejiang, China) at 0.02 mL/g twice a week, and mice in the SMI and PD-1+SMI groups were i.p. administered SMI (batch number: 2007152; Chiatai Qingchunbao Pharmaceutical Co., Ltd, Hangzhou, Zhejiang, China) at 0.02 mL/g twice a week. The administration was stopped after 5 weeks in survival experiment and tumor volume measurement experiment, the survival period of NCI-H226 mice was continued to be observed and recorded until the mice in the IgG groups were all death. Tumor volumes were assessed twice a week and calculated as (length × width^2^)/2.

Mice were anesthetized after the final drug administration in the tumor volume measurement experiment. Anticoagulated whole blood was collected to analyze the number and percentage of peripheral blood leukocytes, while the upper plasma was used for subsequent biochemical analysis. Simultaneously, the left thigh of the mouse was dissected, and the bone marrow cells in the thigh were flushed out using 3 mL of PBS and counted using an automated cell counter (Countess II, Thermo Fisher Scientific, Waltham, MA, USA). The tumors were weighed after separation and placed on a white background to obtain photos at identical heights.

### Biochemical analysis

In mouse model, the plasma biochemical indicators including alanine aminotransferase (ALT) and aspartate aminotransferase (AST) were analyzed by a Cobas C8000 Automatic Biochemical Analysis System (Roche, Basel, Switzerland).

### Hematoxylin–eosin (HE) staining

The heart, liver, spleen, lung, and kidney tissues fixed in 10% formalin solution were embedded in paraffin, sectioned at a thickness of 4 μm, and then stained with HE. The pathological changes of each tissue sample were observed under microscope (OLYMPUS BX61, Tokyo, Japan).

### Tumor sample dissociation

Tumor tissues were washed twice with PBS containing 10% BSA (Sigma-Aldrich, St. Louis, MO, USA), minced into 2–4 mm^3^ small pieces, and then dissociated using a Mouse Tumor Dissociation kit in LLC model or Human Tumor Dissociation kit in NCI-H226 model (Miltenyi Biotech, Bergisch Gladbach, Germany). The isolation of tumor cells was performed according to the previously described method [23]. Live cell suspensions were used for flow cytometry assays or single-cell experiments.

### Flow cytometry analysis

Tumor cells from LLC model were resuspended to a density of 1 × 10^6^ cells/100 μL. The cells were first blocked with anti-mouse CD16/32 for 20 min at 4 °C and then stained with PE anti-mouse CD3 (100204, BioLegend, San Diego, CA, USA), APC anti-mouse CD4 (100312, BioLegend), APC anti-mouse CD8a (100712, BioLegend), APC anti-mouse CD19 (152410, BioLegend), APC anti-mouse NK1.1 (108710, BioLegend), FITC anti-mouse CD25 (101908, BioLegend), or PE anti-mouse FOXp3 (126404, BioLegend) purchased from BioLegend (USA). After incubation of 20 min, cells were washed and terminated with 1 mL PBS, and the stained cells were detected by flow cytometry (Accuri™ C6 CSampler, BD Biosciences, San Jose, CA, USA).

### Single-cell library preparation and analysis

According to the protocols of Chromium Next GEM Single Cell 3ʹ Reagent Kits v3 (10 × Genomics, Pleasanton, CA, USA), we constructed the cDNA library and then pooled and sequenced the library on a Novaseq6000 (Illumina, San Diego, CA, USA).

Raw sequencing data quantification were performed as previously described [23]. Seurat R package (version 4.1.0) was used to analyze raw gene expression matrices. Cells with less than 200 unique genes expressed, more than 6,000 unique genes expressed, more than 10% of reads mapping to mitochondria, or more than 0.1% of reads mapping to hemoglobin had been excluded. Doublets were filtered out using "DoubletFinder" R package (2.0.3). The gene expression matrices of the remaining 38,010 cells were normalized as previously described [23]. The "FindVariableGenes" function was used to select variably expressed genes. Then, the function of "IntegrateData" was used to combine all samples and the function of "ScaleData" was used to scale the integrated data. Cell cycle scoring and regression were performed to remove cell cycle effects on the data analysis, using online method of Seurat (https://satijalab.org/seurat/articles/cell_cycle_vignette.html). Via principal component analysis, the most significant 30 principal components were used to perform uniform manifold approximation and projection (UMAP) dimensionality reduction. The "FindClusters" function was used to perform cell clustering in Seurat, and we annotated the clusters by the expression of canonical marker genes.

### Infer copy number variation (CNV) analysis

The CNV evaluation of each cell was performed by inferCNV R package (version 1.10.1). The CNVs of epithelial cells were calculated and the T and NK cells were applied as the references. The parameters and default Bayesian latent mixture model of inferCNV analysis were performed as previously described [24].

### Inference of developmental trajectory

The normalized gene expression matrix was used as an input to Monocle 3 to deduce the potential lineage differentiation trajectory [25]. The function of "new_cell_data_set" was used to create a new object of Monocle 3, and the function of "preprocess_cds" was used to process the new object with the default parameters. We reduced dimensionality of data using the "reduce_dimension" function before learning the trajectory and ordering cells.

### Transcription factors (TFs) regulatory network analysis

Single-cell regulatory network inference (SCENIC) approach was used to construct gene regulatory network from scRNA-seq data [26]. Briefly, co-expression modules between TFs and the potential target genes were identified, and potential targets which the motif of the corresponding TFs is notably enriched were used to infer the direct target genes. A TF and its direct target genes were defined as a regulon. Regulon Activity Scores was calculated through the area under the recovery curve. Jensen-Shannon Divergence was used to calculate cell-type specific scores of regulators [27].

### Biological processes scoring

Single cells were scored for the expression of gene signatures representing some biological processes. For all biological signatures in this study, function scores were defined as the average normalized expression of the corresponding genes [28].

### Metascape analysis

The top 100 upregulated differentially expressed genes (DEGs) of each cluster were used to perform Metascape analysis [29]. Several databases were used in the Metascape pathway enrichment analysis, including Gene Ontology (GO), Kyoto Encyclopedia of Genes and Genomes (KEGG), Reactome, and MSigDB.

### Survival analysis

RNA-seq and clinical data of LUSC patients (dataset ID: TCGA-LUSC.htseq_counts.tsv, *n* = 550) were obtained from The Cancer Genome Atlas (TCGA) using cgdsr R package. The tumor samples were divided into two groups along with low (25%) and high (75%) target gene expression for all patients. The Kaplan–Meier formula in R package "Survival" was used to perform survival analysis and the ggsurvplot function of the R package "survminer" was used to visualize the survival curve.

### Cell–cell communication analysis

CellPhoneDB was used to analyze cell–cell communications [30]. The genes which expressed in less than 10 cells were filtered out and the top10000 variable genes of filtered gene expression matrix was imported into CellphoneDB. Ten thousand times permutation tests were used to provide the significance and default was used on other method parameters. The ligand-receptor pairs that with no significant mean among all pairs of cell subsets were filtered out.

CellTalkDB (version 0.0.1.6) is a cell communication analysis tools based on database of ligand-receptor interaction pairs in human and mouse [31]. To perform cell–cell communication analysis, the gene expression matrix of high variable genes was imported into CellTalkDB. Ligand-receptor pairs that score less than 0.1 were filtered out.

### Assessing Granzyme A (GzmA) production by NK cells

Human peripheral blood mononuclear cells were purchased from OriBiotech (Shanghai, China). NK cells were isolated using human NK cell isolation kit (130–092-657, Miltenyi Biotec, Bergisch Gladbach, Germany) following the manufacturer’s protocol. Freshly isolated NK cells were cultured with interleukin (IL)-2 (100 ng/ml; C013, Novoprotein, Shanghai, China) for 24 h. Stimulated NK cells were then seeded into 96-well plates (1 × 10^5^ cells/well) and co-cultured with HCI-H226 cells (1 × 10^4^ cells/well) for 24 h following four individually treatments: anti-PD-1 inhibitor monotherapy (10 μg/mL; S007468, Merck Sharp & Dohme Corp, New Jersey, USA), SMI monotherapy (batch number: 2007152; 150 μL/mL; Chiatai Qingchunbao Pharmaceutical Co., Ltd, Hangzhou, Zhejiang, China), combination of anti-PD-1 inhibitor and SMI, and isotype-matched control antibody (10 μg/mL; BP0297, BioXcell, New Hampshire, USA). The GzmA levels in the culture supernatants were examined using enzyme-linked immunosorbent assay (ELISA) kits (69–98755, Mskbio, Wuhan, China) according to the manufacturers’ instructions.

### Immunofluorescence

The tumor tissues fixed in 10% formalin solution were embedded in paraffin, sectioned at a thickness of 3 μm. The slides were washed, permeabilized, and then blocked followed by incubating with primary antibodies at 4 °C overnight. The primary antibodies were human anti-CD3 (17617–1-ap, Proteintech Group, Chicago, IL, USA), human anti-GZMA (K004378P, Solarbio, Beijing, China), human anti-CMC1 (sc-398483, Santa Cruz Biotechnology, CA, USA). After washing, secondary antibodies were used to incubate the sections for 2 h at room temperature. 3D HISTECH (Pannoramic SCAN, Budapest, Hungary) was used to scanning and take images.

### Statistical analysis

Log-rank (Mantel-Cox) tests were used to compare the survival curves and unpaired two-sided t-test was performed to detect the significance between groups. Statistical significance was defined as *P* < 0.05. scRNA-seq analyses and graph generation were performed in R (version 4.1.0).

## Results

### Combination therapy of SMI and PD-1 inhibitor improves the therapeutic effect, prolongs survival, alleviates side effects, and increases tumor-infiltrating NK cells in LLC mouse model.

**Fig. 1:**
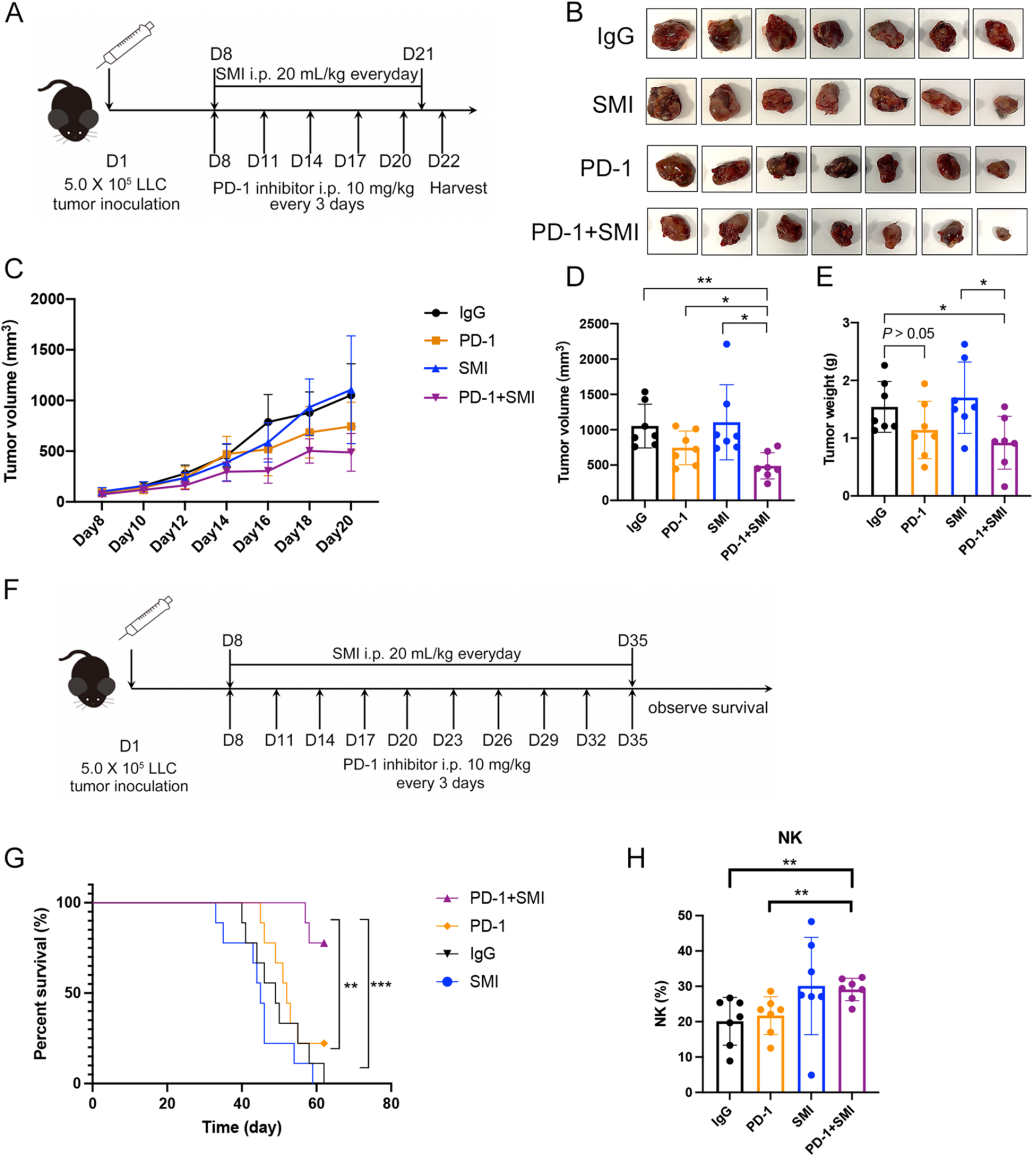
Combination therapy alleviated tumor growth, prolonged survival, and increased tumor-infiltrating NK cells in the LLC mouse model. (**A**) The schedule of tumor volume measurement experiment in the LLC mouse model. (**B**) Photographs of tumors after the different treatments on Day 22. IgG: immunoglobulin G isotype control; PD-1: PD-1 immune-checkpoint blockade antibody; SMI: SMI monotherapy; PD-1+SMI: combination of anti-PD-1 and SMI. (**C**) The changes of tumor volume depicted in (**A**) during different treatments. (**D**) The tumor volume of different treatments on Day 20 (*n* = 7). ^*^P < 0.05, ^**^P < 0.01. (**E**) The tumor weight of different treatments on Day 22 (*n* = 7). ^*^P < 0.05. (**F**) The schedule of survival experiment in the LLC mouse model. (**G**) Percentage of survival of mouse during different treatments (*n* = 7). ^**^P < 0.01, ^***^P < 0.001. (**H**) The proportion of tumor-infiltrating NK cells in the different treatments (*n* = 7). ^**^P < 0.01

To determine whether PD-1 immune-checkpoint blockade combined with the immunomodulator SMI is more effective than PD-1 inhibitor monotherapy in treating NSCLC, the LLC subcutaneous C57BL/6 mouse model was first adopted to detect the efficacy and safety of this combined therapy. Compared with that in the IgG group, tumor growth was significantly inhibited in the PD-1+SMI group (*P* < 0.01), and tumor volume was reduced in the PD-1 group, however, the difference was not significant (Fig. 1A-D). The tumor volume was also significantly reduced in the PD-1+SMI group compared with that in the PD-1 group and SMI group (*P* < 0.05, Fig. 1D). Similarly, the tumor weight on day 22 exhibited the same trend in these groups (Fig. 1E), suggesting that the combined therapy increased antitumor efficacy compared with that in the PD-1 inhibitor monotherapy against LLC in mice.

Moreover, combination therapy significantly prolonged survival of the LLC model mice (Fig. 1F, G). Specifically, 62 days after tumor transplantation, all mice in the IgG and SMI groups died. In contrast, the survival rate in the PD-1 group was 22.2%, while that in the PD-1+SMI group was 77.8%. The combination therapy of PD-1 inhibitor and SMI increased median survival (undefined) compared with that in the IgG, PD-1, and SMI groups (49, 52, and 45 days, respectively). The survival curve of the SMI or PD-1 group was not significantly different compared with that in the IgG group. In contrast, the combination therapy markedly prolonged survival compared with that in the IgG (*P* < 0.001) and PD-1 (*P* < 0.01) groups, suggesting that combined therapy extended survival against LLC compared with that in PD-1 inhibitor monotherapy in mice.

PD-1 inhibitors non-specifically activate the immune system, destroy immune homeostasis, and produce severe inflammatory adverse reactions [32]. The histopathological changes of the main organs of the LLC model mice were studied by HE staining. The results showed that hepatitis and pneumonia were occasionally observed in the PD-1 group, but almost never in the PD-1+SMI group (Additional file 1: Fig. S1A and B), indicating that the combination of SMI and PD-1 inhibitors reduced adverse reactions. The biochemical indicators of AST in individual mouse of PD-1 inhibitor monotherapy increased, while combination therapy of SMI and PD-1 inhibitor could alleviate this phenomenon (Additional file 1: Fig. S2). Other biochemical indices did not show any significant differences between the treatment groups (Additional file 1: Fig. S2 and S3).

As TMEs play an important part in recognizing and killing cancer cells, we further explored the tumor-infiltrating immune cell subsets in the LLC model. SMI adjuvant PD-1 inhibitor treatment significantly increased the proportion of tumor-infiltrating NK cells in the LLC tumor-bearing mice, compared with that in the PD-1 and IgG groups. No significant differences were detected in the proportion of CD8^+^ T cells, CD4^+^ T cells, B cells, and Tregs among different treatment groups (Fig. 1H and Additional file 1: Fig. S1C).

### Combination therapy of SMI and PD-1 inhibitor sensitizes the antitumor effect of the PD-1 inhibitor and moderates side effects in a humanized mouse model

**Fig. 2:**
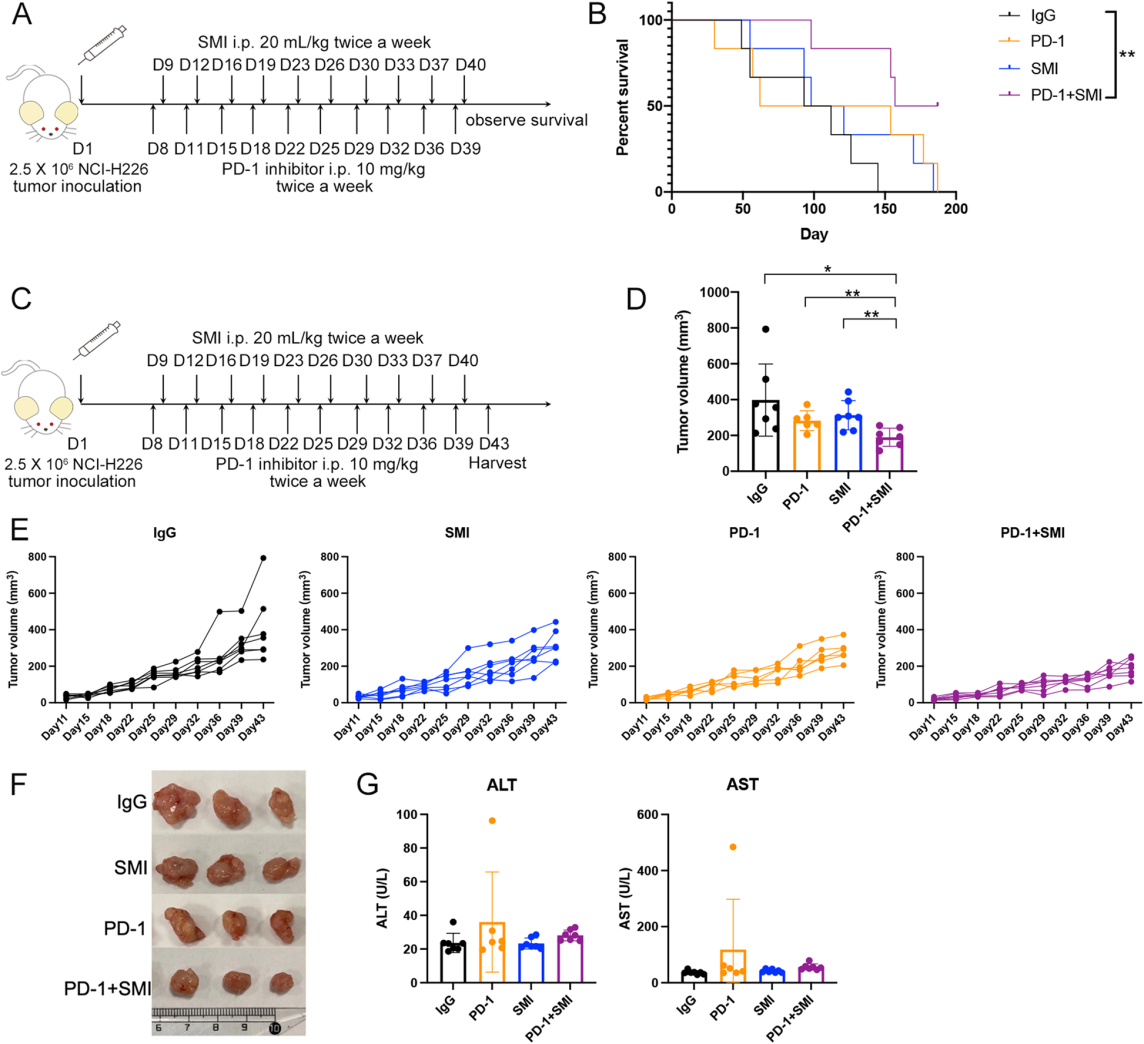
Combination therapy strengthened the antitumor effect and alleviated side effects in the humanized mouse model. (**A**) The schedule of survival experiment in the humanized mouse model. IgG: immunoglobulin G isotype control; PD-1: PD-1 immune-checkpoint blockade antibody; SMI: SMI monotherapy; PD-1+SMI: combination of anti-PD-1 and SMI. (**B**) Percentage of survival of mouse during different treatments (*n* = 7). ^**^P < 0.01. (**C**) The schedule of tumor volume measurement experiment in the humanized mouse model. (**D**) The tumor volume of different treatments on Day 43. ^*^P < 0.05, ^**^P < 0.01. A mouse in the PD-1 group died on Day 32. IgG, SMI, and PD-1 + SMI groups, *n* = 7; PD-1 group, *n* = 6. (**E**) The changes of tumor volume depicted during different treatments. (**F**) Representative photographs of tumors after the different treatments on Day 43. (**G**) The alanine aminotransferase (ALT) and aspartate aminotransferase (AST) in the different treatments

We further assessed the efficacy of the combination therapy in the HuNCG mouse NSCLC model with a humanized immune system. As shown in Fig. 2A and 2B, all mice in the IgG group died within 145 days of receiving the tumor, while all mice in the PD-1 and SMI groups died within 187 and 184 days, respectively. Notably, 50% of the mice in the combination group survived throughout the observation period. The combination therapy prolonged mouse survival compared with that in the IgG and PD-1 groups. Consistently, the mice that received combination therapy developed smaller tumors compared with those in the IgG group, as reflected in their lower tumor volume (Fig. 2C-F and Additional file 1: Fig. S4). No significant differences were detected between the PD-1 and IgG groups in these measurements. These results suggest that anti-PD-1 alone does not provide an effective response against NSCLC in the HuNCG mouse model, however, PD-1 inhibitor combined with SMI may increase the anti-NSCLC response.

Furthermore, we evaluated the irAEs after combination therapy and anti-PD-1 monotherapy. First, compared with that in the IgG group, individual mice in the PD-1 group displayed higher levels of ALT and AST, which was not observed in the combination treatment (Fig. 2G). Second, the results of HE staining showed that moderate amount of small focal infiltration of inflammatory cells were found in the liver of PD-1 inhibitor monotherapy treated mice, as well as more inflammatory cell infiltration and severe thickening of the local alveolar wall were found in the lung of PD-1 inhibitor monotherapy treated mice, while combination therapy could alleviate this phenomenon (Additional file 1: Fig. S5 and S6). This suggests that the function of the liver and lung were not affected by the combination treatment and SMI may reduce hepatitis and pneumonia induced by anti-PD-1 treatment. The number of bone marrow cells and percentage of peripheral blood lymphocytes, white blood cells, neutrophils, monocytes, eosinophils, and basophils were not significantly different between the treatment groups (Additional file 1: Fig. S7).

### Single-cell transcriptional landscape in the LUSC tumor in a humanized mouse model under different drug treatments

**Fig. 3:**
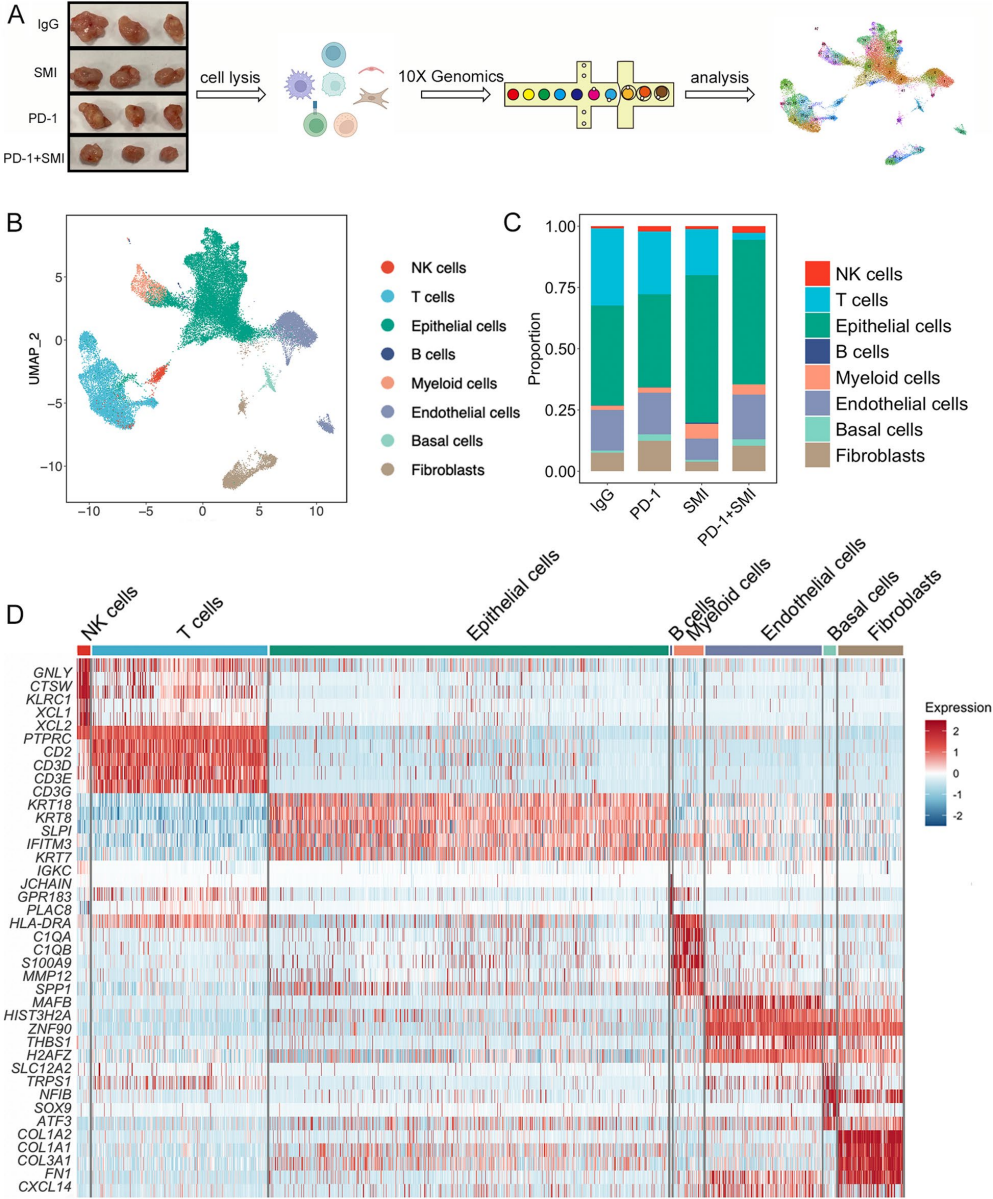
Single-cell transcriptome profiles of four treatments in the humanized mouse model. (**A**) The overview of single-cell RNA sequencing (scRNA-seq) experiment. Tumor tissues from three representative mice in each group were collected. IgG: immunoglobulin G isotype control; PD-1: PD-1 immune-checkpoint blockade antibody; SMI: SMI monotherapy; PD-1+SMI: combination of anti-PD-1 and SMI. (**B**) Uniform manifold approximation and projection (UMAP) showing eight cell types for 38,010 cells. (**C**) Proportion of eight cell types in the IgG, PD-1, SMI, and PD-1+SMI. (**D**) Heatmap showing differentially expressed genes (DEGs) for eight cell types

To depict the transcriptional landscape and identify the synergistic mechanisms of combined PD-1 inhibitor and SMI therapy, we performed scRNA-seq analysis on the tumor tissues from three representative mice in each group using 10X Genomics (Fig. 3A). After quality filtering, 38,010 cells were detected, among which 10,065, 12,678, 9,786, and 5,481 cells were collected from the IgG, SMI, PD-1, and PD-1+SMI groups, respectively. After unsupervised cell clustering and UMAP dimensionality reduction, eight distinct lineages, including epithelial (18,681; *KRT8*, *KRT18*, and *KRT19*), basal (583; *SOX9*, *KRT14*, and *TPRS1*), endothelial (5,440; *RAMP2*, *MAFB*, and *PICALM*), fibroblast (3,034; *COL1A1*, *COL1A2*, and *ACTA2*), T (8,218; *CD3D*, *CD3E*, and *CD3G*), B (68; *CD79A*, *JCHAIN*, and *MS4A1*), NK(604; *KLRD1*, *NCAM1*, and *PRF1*), and myeloid (1,382; *C1QA*, *C1QB*, and *LYZ*) cells, were identified based on marker gene expression (Fig. 3B-D, Additional file 1: Fig. S8A-B, and Additional file 2: Table S1). The number of B cells in the tumor of the humanized mouse model was extremely low, which may be because B cells from the humanized mice were mainly immature and B cell function was impaired [33]. The proportion of each cell lineage varied greatly between the groups (Fig. 3C), suggesting that different cell subpopulations infiltrated the tumor under different drug treatments and reacted to the tumor via distinct mechanisms.

### Relationship between the seven subpopulations of malignant epithelial cells and different drug treatments

**Fig. 4:**
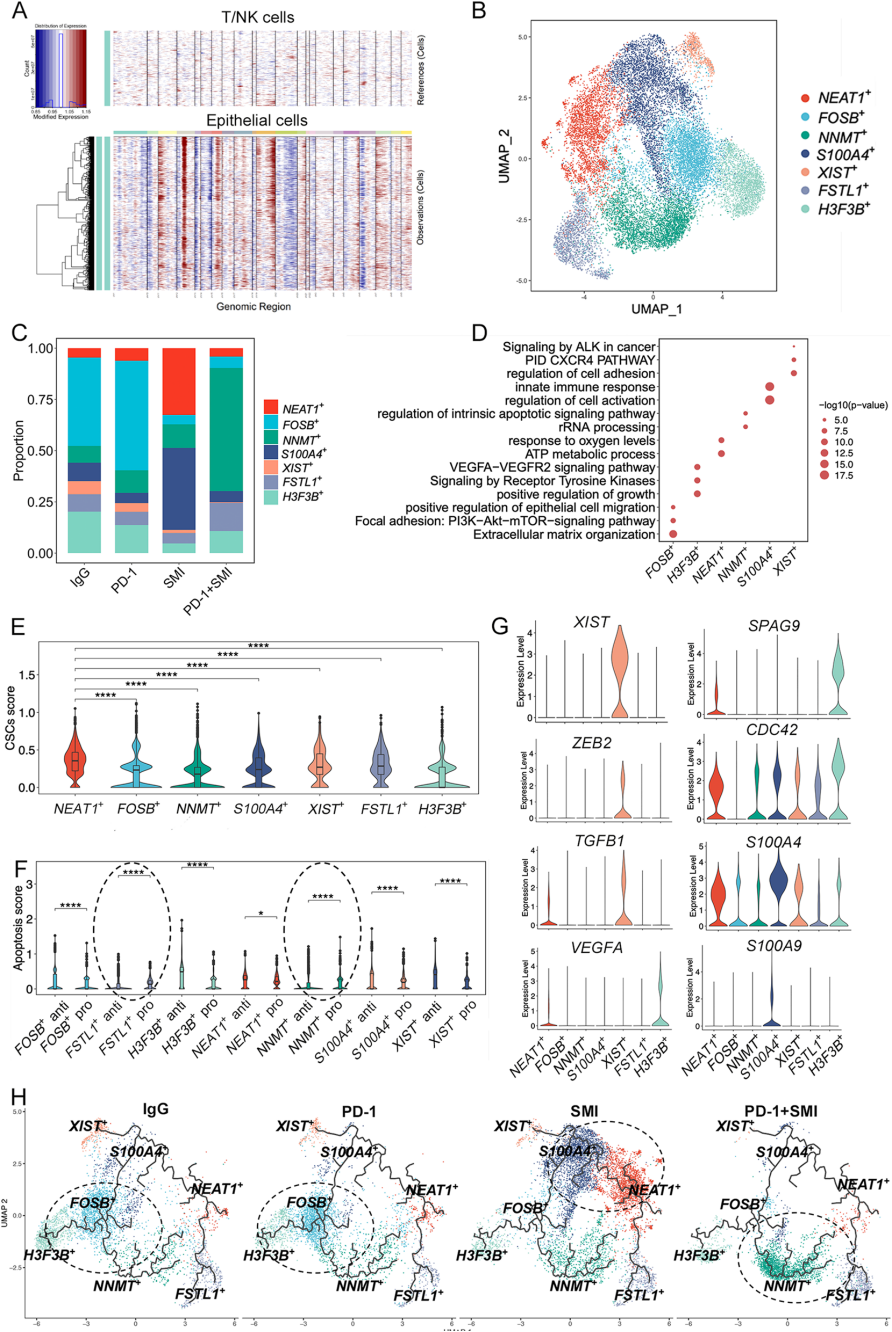
Seven subclusters of malignant epithelial cells were identified among different treatments. (**A**) Malignant epithelial cells were distinguished with T/NK cells by inferred large-scale chromosomal copy number variations (CNVs). (**B**) UMAP of the seven malignant epithelial clusters: *NEAT1*^+^ tumor cells (*NEAT1*^+^), *FOSB*^+^ tumor cells (*FOSB*^+^), *NNMT*^+^ tumor cells (*NNMT*^+^), *S100A4*^+^ tumor cells (*S100A4*^+^), *XIST*^+^ tumor cells (*XIST*^+^), *FSTL1*^+^ tumor cells (*FSTL1*^+^), *H3F3B*^+^ tumor cells (*H3F3B*^+^). (**C**) Relative proportion of seven malignant epithelial clusters in four different treatments. IgG: immunoglobulin G isotype control; PD-1: PD-1 immune-checkpoint blockade antibody; SMI: SMI monotherapy; PD-1+SMI: combination of anti-PD-1 and SMI. (**D**) Dot plot showing the top representative biological pathways of seven malignant epithelial clusters based on DEGs using Metascape analysis. (**E**) Violin plot showing the highest cancer stem cells (CSCs) score in *NEAT1*^+^ tumor cells compared with that in other subclusters. ^****^P < 0.0001. (**F**) Violin plot showing the mean score of the apoptosis or anti-apoptosis signature across seven malignant epithelial clusters. *NNMT*^+^ and *FSTL1*^+^ tumor cells displayed significantly higher pro-apoptosis scores than anti-apoptosis score, while other subcluster cells displayed significantly higher anti-apoptosis scores than pro-apoptosis scores. ^*^P < 0.05, ^****^P < 0.0001. (**G**) Violin plot showing expression of *XIST*, *ZEB2*, *TGFB1*, *VEGFA*, *SPAG9*, *CDC42*, *S100A9*, and *S100A4* in seven malignant epithelial clusters. (H) Unsupervised transcriptional trajectory of seven malignant epithelial clusters predicted by Monocle 3 in four different treatments

As the epithelial cells in the LUSC tumor were developed from human LUSC NCI-H226 cell lines, the epithelial cells in the tumor were considered malignant [34]. Consistently, the cytokeratin tumor markers, *KRT8*, *KRT18*, and *KRT19*, were most abundantly expressed in these epithelial cells (Additional file 1: Fig. S9A) [35]. Moreover, we found that these epithelial cells experienced significantly higher CNV levels compared with that in the reference cells (Fig. 4A), demonstrating that these cells were malignant.

Clustering analysis of malignant cells in the four treatment groups revealed seven subclusters (Fig. 4B and Additional file 1: Fig. S9B), and transcriptional heterogeneity was detected in these malignant cells. *FOSB*^+^ tumor cells were found mainly in the PD-1 and IgG groups (Fig. 4C). Upon Metascape analysis, *FOSB*^+^ tumor cells were found to show characteristics of extracellular matrix (ECM) organization, focal adhesion, and positive regulation of epithelial cell migration, based on the upregulated DEGs between the *FOSB*^+^ tumor cells and other malignant epithelia (Fig. 4D and Additional file 1: Fig. S10A). *SPP1*, *ITGA3*, and *VTN*, which are related to cell adhesion and tumor cell invasion and migration promotion, were clustered according to the aforementioned pathways (Additional file 2: Table S2) [36]. Other genes that promote lung cancer cell invasion and metastasis, including *CDK6*, *DNAJB6*, *ATF4*, *STAT1*, and *HIF1A*, were also clustered in the aforementioned pathways (Additional file 2: Table S2) [37, 38]. Therefore, *FOSB*^+^ tumor cells may possess invasive and migrative properties. Another subcluster, *NEAT1*^+^ tumor cells, were mainly found in the SMI group (Fig. 4C). Metascape analysis revealed that the ATP metabolic process and response to oxygen levels pathway were enriched in *NEAT1*^+^ tumor cells (Fig. 4D and Additional file 1: Fig. S10A), where oxidative phosphorylation (OXPHOS)-related genes, *COX1*, *COX2*, *COX3*, *ND1*, *ND2*, *ND4*, and *ATP6*, were clustered (Additional file 2: Table S2). Cancer stem cells (CSCs) exhibit OXPHOS dependency and *NEAT1* also functions as an inducer of CSC-like phenotypes in NSCLC [39]. The CSCs score, represented by the expression of marker genes for CSCs in NSCLC (*NANOG*, *POU5F1* [*CD90*], *ABCB1*, *ABCG2*, *ALDH1A1*, *CD24*, *CD44*, *CXCR4*, *PROM1* [*CD133*], *EPCAM*, *ICAM1*, *ALCAM*, *MYC*, and *NES*) [40, 41], was significantly upregulated in *NEAT1*^+^ tumor cells compared with that in other subclusters, suggesting that *NEAT1*^+^ tumor cells present a CSCs phenotype (Fig. 4E and Additional file 1: Fig. S9C). *NNMT*^+^ tumor cells were the main cell type identified in the PD-1+SMI group (Fig. 4C) and regulation of the intrinsic apoptotic signaling pathway was enriched in this subcluster as analyzed by Metascape (Fig. 4D and Additional file 1: Fig. S10A). *VDAC2*, *GADD45A*, and *RPL26*, which mediate apoptosis and limit tumor development, were enriched in the aforementioned pathways (Additional file 2: Table S2) [42–44]. Intrinsic pro- and anti-apoptotic scores for seven subclusters were calculated based on the expression of genes related to lung cancer cell intrinsic pro-apoptosis (*BAX*, *BAK1*, *BAD*, *BIK*, *BID*, *BCL2L11*, *HRK*, *BBC3*, and *PMAIP1*) or anti-apoptosis (*BCL2*, *BCL2L1*, *BCL2L2*, *BCL2A1*, and *MCL1*) [45]. As shown in Fig. 4F, *NNMT*^+^ and *FSTL1*^+^ tumor cells displayed significantly higher pro-apoptosis scores than anti-apoptosis score, while other subcluster cells displayed significantly higher anti-apoptosis scores than pro-apoptosis scores, suggesting that *NNMT*^+^ and *FSTL1*^+^ tumor cells may be susceptible to apoptosis. However, *FSTL1*^+^ tumor cells, which were most abundant in the PD-1+SMI group (Fig. 4C), expressed high levels of the lung CSCs marker genes *CD44* and *ALCAM* (Additional file 1: Fig. S9D), and the CSCs score was significantly higher than those in the other subclusters, except for *NEAT1*^+^ and *XIST*^+^ tumor cells (Additional file 1: Fig. S9E). This indicates that *FSTL1*^+^ tumor cells may act as CSCs with high apoptosis potential. *XIST*^+^ tumor cells were most abundant in the IgG group (Fig. 4C), in which regulation of cell adhesion, the CXCR4 pathway, and ALK signaling in cancer were enriched (Fig. 4D and Additional file 1: Fig. S10A). The lung cancer epithelial-mesenchymal transition (EMT)-related genes, *CXCR4*, *TGFB1*, *EML4*, and *TNFAIP3* (*A20*), were clustered in these aforementioned pathways (Additional file 2: Table S2) [46, 47]. A previous study demonstrated that *XIST* promotes transforming growth factor-beta (TGF-β)-induced EMT which may regulate the miR-367/miR-141-ZEB2 axis in NSCLC [48], and our results showed that *ZEB2* was highly expressed in the *XIST*^+^ tumor cells (Fig. 4G). This suggests that EMT progression was induced in *XIST*^+^ tumor cells. *H3F3B*^+^ tumor cells were primarily present in the IgG group (Fig. 4C), in which the vascular endothelial growth factor (VEGF) A-vascular endothelial growth factor receptor (VEGFR) 2 signaling, positive regulation of growth, and receptor tyrosine kinase signaling pathways were enriched (Fig. 4D and Additional file 1: Fig. S10A). Essential genes in angiogenesis and invasion, *VEGFA*, *SPAG9*, and *CDC42*, were distinctly upregulated in *H3F3B*^+^ tumor cells and clustered in the angiogenesis and invasion pathways (Fig. 4G and Additional file 2: Table S2), suggesting that *H3F3B*^+^ tumor cells may exert angiogenic and invasive effects. *S100A4*^+^ tumor cells were highly populated in the SMI group (Fig. 4C), and the innate immune response and regulation of cell activation were enriched in this group (Fig. 4D and Additional file 1: Fig. S10A). This subcluster highly expressed *S100A9* and *S100A4* (Fig. 4G). *S100A4* is a key element related to EMT signal that induces cell invasion and proliferation in NSCLC, while *S100A9* is a key factor in the immune response and inflammation modulation related to poorly differentiated NSCLC [49]. Therefore, *S100A4*^+^ tumor cells may be associated with the immune response and EMT.

Furthermore, we performed pseudotime analyses on trajectory deductions derived from Monocle3 to comprehensively understand the relationship between the characteristics of tumor cells and different treatments. Specifically, *NEAT1*^+^ tumor cells with the CSCs phenotype were observed in the initial portion, followed by two separate trajectory branches: apoptotic CSCs *FSTL1*^+^ tumor cells and EMT tendency and immune-associated *S100A4*^+^ tumor cells (Additional file 1: Fig. S10B). Next, another two trajectory branches, EMT-induced *XIST*^+^ tumor cells and invasive and migrative *FOSB*^+^ tumor cells, were separated after *S100A4*^+^ tumor cells. Finally, angiogenic and invasive *H3F3B*^+^ tumor cells and apoptotic *NNMT*^+^ tumor cells appeared at the most distant part of the trajectory. As shown in Fig. 4H, tumor cells from the IgG and PD-1 groups were mainly clustered in the invasive and migrative branches (*H3F3B*^+^ and *FOSB*^+^ tumor cells), while cells from the SMI group were mainly clustered in the CSCs and EMT branches (*NEAT1*^+^ and *S100A4*^+^ tumor cells). Lastly, cells from the combination therapy PD-1+SMI group were mainly clustered in the apoptotic branch (*NNMT*^+^ and *FSTL1*^+^ tumor cells), suggesting that PD-1 inhibitor combined with SMI induces tumor apoptosis.

A LUSC cohort from TCGA dataset (TCGA-LUSC.htseq_counts.tsv, *n* = 550) was used to perform the survival analysis of cancer cell-associated genes. Patients with LUSC showing high expression of *FOSB*^+^ tumor cell-associated genes (*FSTL3* and *VTN*), *NEAT1*^+^ tumor cell-associated genes (*PAPPA*, *CCN1*, and *RRAD*), and *XIST*^+^ tumor cell-associated genes (*GPRIN3*) exhibited lower overall survival (*P* < 0.05) than those with low expression, whereas patients with LUSC showing high expression of *NNMT*^+^ tumor cell-associated genes (*ERH*) exhibited higher overall survival (*P* < 0.05, Additional file 1: Fig. S10C) than those with low expression.

To further understand the characteristics of the tumor cells, potential regulons (TFs and their target genes) were inferred using SCENIC analysis. The top 10 TFs in each subcluster are shown in Additional file 2: Table S3 and Additional file 1: Fig. S11. TFs in *XIST*^+^ and *S100A4*^+^ tumor cells were similar, particularly RUNX3, REL, STAT4, and E2F1, which regulate the EMT process. RELB, NFKB1, BCL3, and FOXO3, found in *NEAT1*^+^ tumor cells, were closely related to the CSCs phenotype [50]. CTCF, FOS, ATF2, and YY1 in *H3F3B*^+^ tumor cells were found to regulate angiogenesis [51]. Furthermore, TFE3, TFDP1, NFYB, and RB1, associated with the cell cycle and apoptosis [52], were inferred in *FSTL1*^+^ tumor cells. Lastly, POLE3, which is associated with the differentiation of lymphocyte-like cells [53], was inferred in *NNMT*^+^ tumor cells. These TFs may provide new insights into therapies for LUSC.

### Combination therapy of SMI and PD-1 inhibitor increases the proportion of *GZMA*^high^ NK cells and decreases the proportion of exhausted T cells

**Fig. 5:**
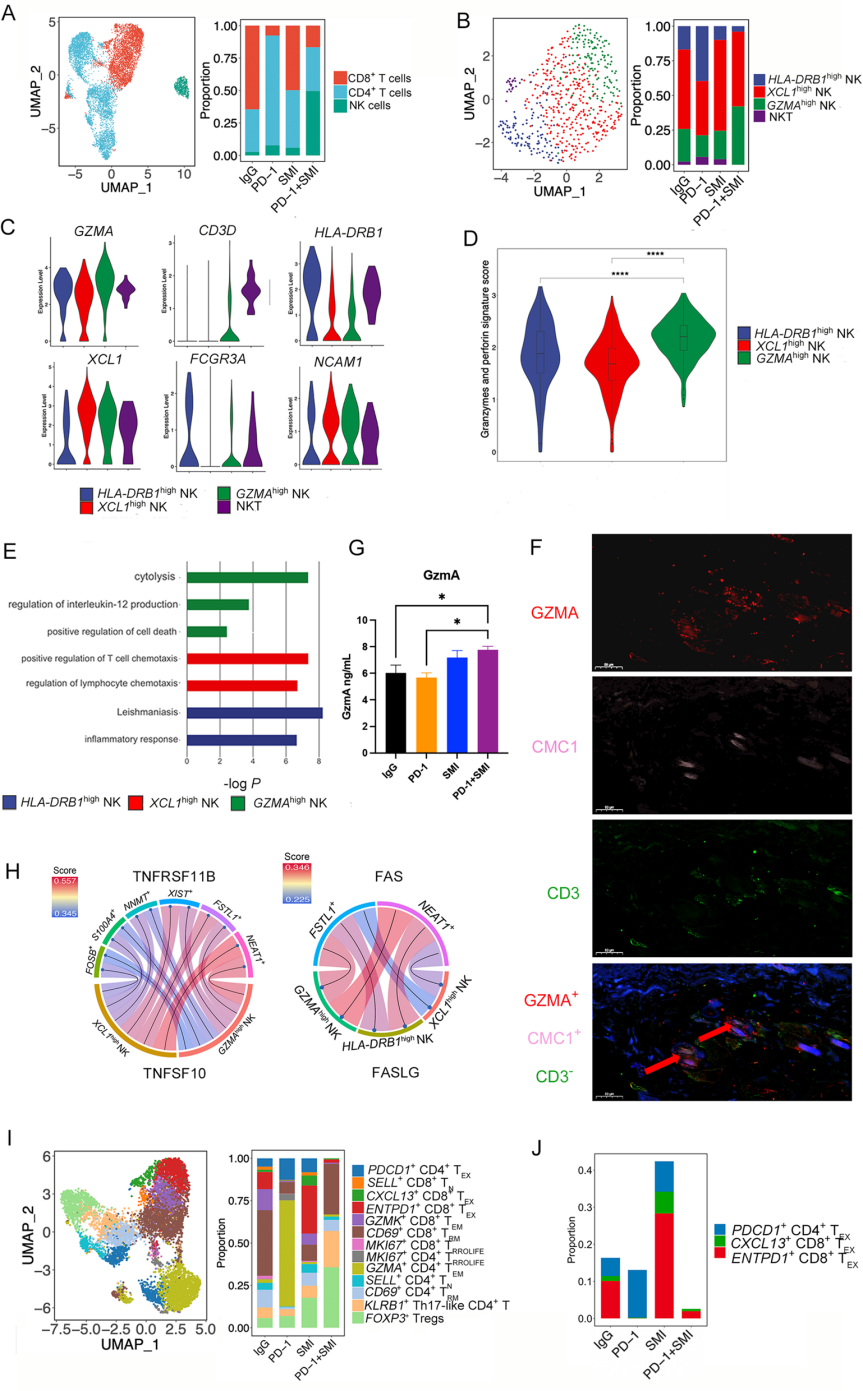
The proportion of *GZMA*^high^ NK cells was increased and the proportion of exhausted T cells was decreased in the combination therapy. (**A**) UMAP of CD4^+^ T cells, CD8^+^ T cells, and NK cells were showed on the left, and relative proportion of these three clusters in four different treatments were showed on the right. (**B**) UMAP of *GZMA*^high^ NK, *HLA-DRB1*^high^ NK, *XCL1*^high^ NK, and NKT cells were showed on the left, and relative proportion of these four clusters in four different treatments were showed on the right. (**C**) Violin plot showing expression of *GZMA*, *CD3D*, *HLA-DRB1*, *XCL1*, *NCAM1*, and *FCGR3A* in four NK clusters. (**D**) Violin plot showing the highest granzyme and perforin signature score in *GZMA*^high^ NK cells compared with that in other subclusters. ^****^P < 0.0001. (**E**) Metascape analysis showing the top representative biological pathways of three NK subclusters based on DEGs. (**F**) Immunofluorescence staining indicates the co-expression of GZMA, CMC1, and DAPI (nuclei) on *GZMA*^high^ NK cells in PD-1+SMI group. (**G**) Granzyme A levels in the co-culture of NK cells and LUSC NCI-H226 cells following different treatment. ^*^P < 0.05, *n* = 2. (**H**) Chord diagram showing the ligand-receptor pair TNFSF10-TNFRSF11B involved in interactions between *GZMA*^high^ NK cells and tumor subclusters (*FSTL1*^+^ tumor cells, *XIST*^+^ tumor cells, *S100A4*^+^ tumor cells, *NNMT*^+^ tumor cells, *FOSB*^+^ tumor cells, and *NEAT1*^+^ tumor cells), and between *XCL1*^high^ NK cells and the same tumor subclusters, also the ligand-receptor FASL-FAS pair was inferred between three NK subclusters and *FSTL1*^+^ tumor cells, and between three NK subclusters and *NEAT1*^+^ tumor cells. (**I**) UMAP of thirteen T cell subclusters were showed on the left. Relative proportion of these T subclusters in different treatments were showed on the right. (**J**) Relative proportion of *CXCL13*^+^ CD8^+^ T_EX_, *ENTPD1*^+^ CD8^+^ T_EX_, and *PDCD1*^+^ CD4^+^ T_EX_ cells in different treatments

T and NK cells play an essential role in mediating antitumor processes in lung cancer. In total, 8,822 T and NK cells were collected and identified as CD4^+^ T cells, CD8^+^ T cells, and NK cells, where NK cells were the most abundant in the PD-1+SMI group (Fig. 5A). NK cells were classified into four subclusters: *GZMA*^high^ NK, *XCL1*^high^ NK, *HLA-DRB1*^high^ NK, and nature killer T (NKT) cells, according to the expression of the DEGs (Fig. 5B-C and Additional file 1: Fig. S12A). *GZMA*^high^ NK, *HLA-DRB1*^high^ NK, and NKT cells were *FCGR3A* (CD16)-positive and *NCAM1* (CD56)-dim, whereas *XCL1*^high^ NK cells were *FCGR3A*-dim and *NCAM1*-bright (Fig. 5C)*.* Transcriptional heterogeneity in NK cells was detected among the different treatment groups (Fig. 5B). NKT cells were mainly found in the PD-1, IgG, and SMI groups (Fig. 5B), whereas no NKT cells were detected in the PD-1+SMI group. Moreover, inhibitory receptors (*PDCD1*, *TIGIT*, *LAG3*, *HAVCR2*, and *CTLA4*) were highly expressed in NKT cells, suggesting that these cells were in an exhausted state (Additional file 1: Fig. S12B), and the PD-1+SMI combination treatment eliminated such exhausted NKT cells. *GZMA*^high^ NK cells were most highly enriched in the PD-1+SMI group (Fig. 5B). One mechanism of NK cell cytotoxicity is the release of granzymes and perforin [54]. Here, we calculated the granzyme and perforin signature scores of the NK subclusters based on the expression of *GZMA*, *GZMB*, *GZMK*, *GZMM*, *GZMH*, *PRF1*, and *GNLY* [54]. The *GZMA*^high^ NK cells demonstrated the highest granzyme and perforin signature scores compared with those in other subclusters (*P* < 0.01, Fig. 5D). Another mechanism of NK cell cytotoxicity occurs by inducing apoptosis in target cells through the interaction between the Fas ligand (FASL)-CD95/Fas or tumor necrosis factor (TNF)-related apoptosis-inducing ligand (TRAIL)-TRAIL-R1/-R2 [54]. *GZMA*^high^ NK cells expressed high levels of both *FASLG* and *TNFSF10* (Additional file 1: Fig. S12C). Moreover, Metascape analysis revealed that cytolysis, regulation of IL-12 production, and positive regulation of cell death were enriched in the *GZMA*^high^ NK cells (Fig. 5E), indicating that *GZMA*^high^ NK cells may exert cytotoxic functions to kill tumor cells. PD-1+SMI combination treatment may enhance this effect by inducing increased infiltration of *GZMA*^high^ NK cells into the tumor. *XCL1*^high^ NK cells expressed a high level of chemokine genes, *XCL1*, *XCL2*, and *TNFSF14* (Additional file 1: Fig. S12A), which are related to the maturation and recruitment of DCs [55, 56]. *FLT3LG*, another cytokine produced by NK cells to regulate DCs levels [57], was highly expressed in *GZMA*^high^ and *XCL1*^high^ NK cells (Additional file 1: Fig. S12C). Positive regulation of T cell chemotaxis and regulation of leukocyte activation were enriched in the *XCL1*^high^ NK cells (Fig. 5E), suggesting that they may be involved in immune cell chemotaxis and activation. *HLA-DRB1*^high^ NK cells were mainly found in the PD-1 group, where inflammatory responses and leishmaniasis were enriched (Fig. 5B and E). Visceral leishmaniasis may be a side effect of ICIs treatment, and human leukocyte antigen (HLA) II activation has been reported in ICIs-induced thyroiditis [58]. Thus, we speculated that *HLA-DRB1*^high^ NK cells participate in the inflammatory response to ICIs-induced inflammation, and PD-1+SMI combination treatment decreased the proportion of *HLA-DRB1*^high^ NK cells compared with that in PD-1 group, which was consistent with the pathological results. In addition, NK cells in the PD-1+SMI group expressed higher level of *GZMA* and *XCL1* compared with those in other groups (Additional file 1: Fig. S12D), indicating that combination therapy increased the granzyme and chemokine expression in NK cells. As shown in Fig. 5F, immunofluorescence staining showed that *GZMA*^high^ NK cells appeared in the PD-1+SMI group, showing this kind of subcluster existed in the NSCLC. Furthermore, we performed an in vitro experiment by co-culturing stimulated NK cells from human peripheral blood mononuclear cells and LUSC NCI-H226 cells to investigate the influence of combination therapy on NK cells. As a result, the amount of GzmA secreted by NK cells was significantly improved in the PD-1+SMI group compared with that in the IgG and PD-1 groups (Fig. 5G), confirming that combination therapy increased expression of GzmA in NK cells.

CCIs among different subclusters in the TME influence cancer progression and affect responses to therapy. Specifically, interactions driving TRAIL-mediated apoptosis via the TNFSF10 (TRAIL)-TNFRSF11B ligand-receptor pair were identified between *GZMA*^high^ NK cells and tumor subclusters (*FSTL1*^+^ tumor cells, *XIST*^+^ tumor cells, *S100A4*^+^ tumor cells, *NNMT*^+^ tumor cells, *FOSB*^+^ tumor cells, and *NEAT1*^+^ tumor cells), and between *XCL1*^high^ NK cells and the same tumor subclusters (Fig. 5H). Furthermore, the FASL-FAS pair was inferred between three NK subclusters and *FSTL1*^+^ tumor cells, and between three NK subclusters and *NEAT1*^+^ tumor cells, while *GZMA*^high^ NK cells presented the highest interacting value (Fig. 5H), indicating that *GZMA*^high^ NK cells may induce apoptosis in tumor cells through death receptor systems [54].

We identified 13 subclusters of T cells, including *SELL*^+^ naïve CD8^+^ (*SELL*^+^ CD8^+^ T_N_), *CXCL13*^+^ exhausted CD8^+^ (*CXCL13*^+^ CD8^+^ T_EX_), *ENTPD1*^+^ exhausted CD8^+^ (*ENTPD1*^+^ CD8^+^ T_EX_), *GZMK*^+^ effector memory CD8^+^ (*GZMK*^+^ CD8^+^ T_EM_), *CD69*^+^ tissue-resident memory CD8^+^ (*CD69*^+^ CD8^+^ T_RM_), *MKI67*^+^ proliferating CD8^+^ (*MKI67*^+^ CD8^+^ T_PROLIFE_), *MKI67*^+^ proliferating CD4^+^ (*MKI67*^+^ CD4^+^ T_PROLIFE_), *GZMA*^+^ effector memory CD4^+^ (*GZMA*^+^ CD4^+^ T_EM_), *SELL*^+^ naïve CD4^+^ (*SELL*^+^ CD4^+^ T_N_), *CD69*^+^ tissue-resident memory CD4^+^ (*CD69*^+^ CD4^+^ T_RM_), *PDCD1*^+^ exhausted CD4^+^ (*PDCD1*^+^ CD4^+^ T_EX_), *FOXP3*^+^ Tregs, and *KLRB1*^+^ CD4^+^ T helper 17-like (*KLRB1*^+^ Th17-like CD4^+^ T cells) T cells, according to the expression of their respective markers (Fig. 5I, Additional file 1: Fig. S12E, and Additional file 2: Table S4) [59, 60]. The proportion of *CXCL13*^+^ CD8^+^ T_EX_, *ENTPD1*^+^ CD8^+^ T_EX_, and *PDCD1*^+^ CD4^+^ T_EX_ cells that highly expressed co-inhibitory immune checkpoint genes (*LAG3*, *TIGIT*, *PDCD1*, *HAVCR2*, and *CTLA4*), were notably decreased in the PD-1+SMI group compared with that in the PD-1, IgG, and SMI groups (Fig. 5J and Additional file 1: Fig. S12E). Although the proportion of Tregs were high in the PD-1+SMI group, the expression of suppressive Tregs markers (*FOXP3*, *IL2RA*, and *IKZF2)* were lower in the PD-1+SMI compared with those in the PD-1 and IgG groups, suggesting that the Tregs in the PD-1+SMI group may be resting (Fig. 5I and Additional file 1: Fig. S12F) [61]. Moreover, the proportion of *KLRB1*^+^ CD4^+^ Th17-like T cells was the highest in the PD-1+SMI group compared with that in the remaining groups (Fig. 5I). *KLRB1*^+^ CD4^+^ Th17-like T cells expressed the Th17 lymphocyte-associated genes, *CCR6* and *RORC* (Additional file 1: Fig. S12G). Cytokine signaling in the immune system, T cell costimulation, and regulation of the defense response were enriched in the *KLRB1*^+^ CD4^+^ Th17-like T cells (Additional file 1: Fig. S12H). The CD4^+^ T memory cell-associated genes, *TNFSF13B*, *TNFSF14*, *TNFSF8*, and *TNFRSF25,* were clustered in the aforementioned pathways (Additional file 2: Table S2), indicating that *KLRB1*^+^ CD4^+^ Th17-like T cells may exert memory functions. Some natural Tregs convert into Th17-like cells and their balance is important in the immune response to tumors [60]. Moreover, the PD-1+SMI group exhibited high cytotoxic and low exhausted gene signature scores in all the T and NK cell populations, suggesting that combination therapy increased the cytotoxic response and decreased the exhausted status of T/NK cells compared with that in monotherapy (Additional file 1: Fig. S12I).

### Combination therapy of SMI and PD-1 inhibitor decreases the proportion of M2-like macrophages

**Fig. 6:**
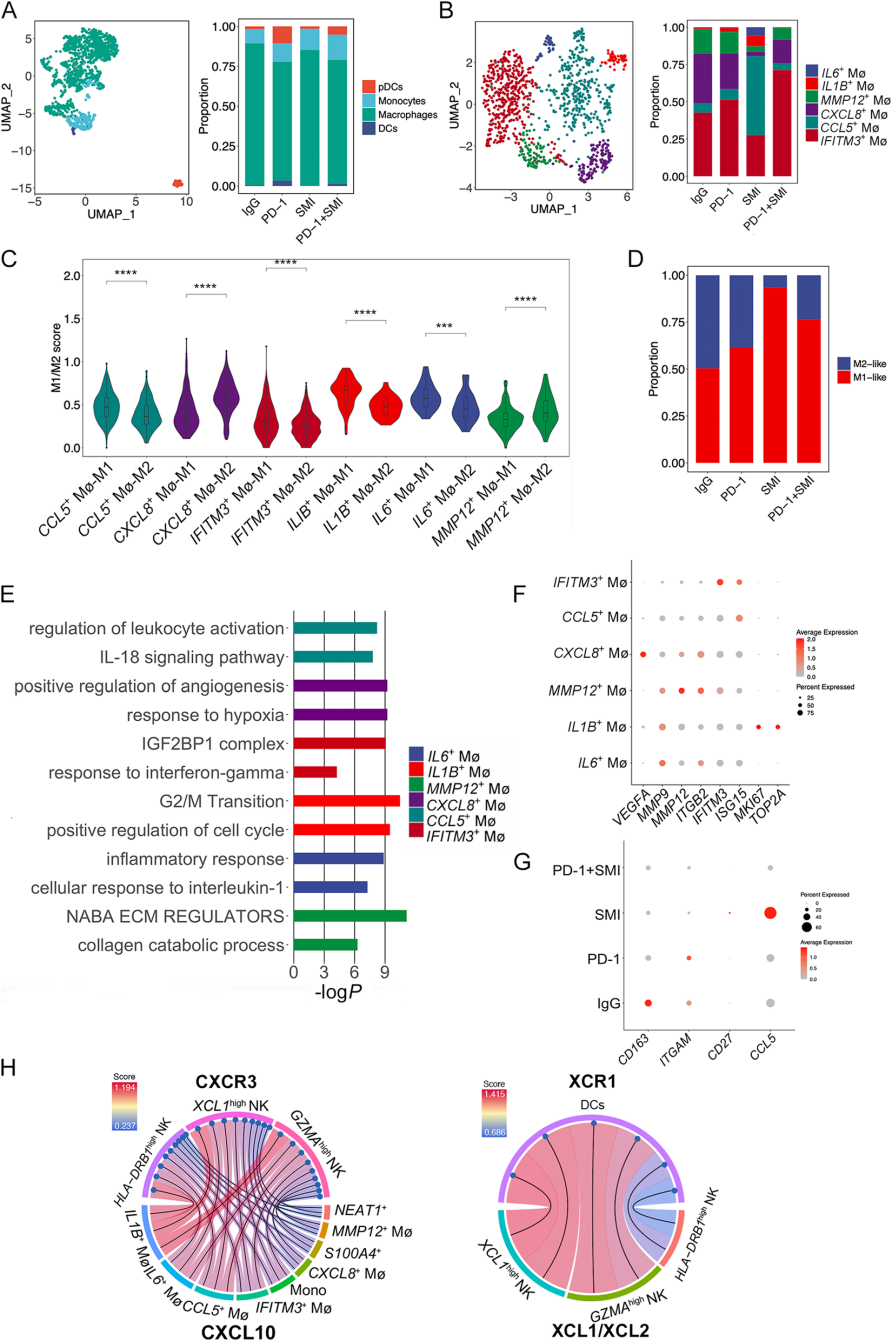
The proportion of M2-like macrophages was decreased in the combination therapy. (**A**) UMAP of macrophages, monocytes, classical DCs, and plasmacytoid DCs, relative proportion of these four clusters in four different treatments were showed on the right. IgG: immunoglobulin G isotype control; PD-1: PD-1 immune-checkpoint blockade antibody; SMI: SMI monotherapy; PD-1+SMI: combination of anti-PD-1 and SMI. (**B**) UMAP of six macrophage subclusters. Relative proportion of these six clusters in four different treatments were showed on the right. (**C**) Violin plot showing the mean score of the M1 or M2 signature across six macrophage subclusters. ^***^P < 0.001, ^****^P < 0.0001. (**D**) Relative proportion of M1 and M2 cells in four different treatments. (**E**) Metascape analysis showing the top representative biological pathways of six macrophage subclusters. (**F**) Dot plot showing expression of *VEGFA*, *MMP9*, *MMP12*, *ITGB2*, *IFITM3*, *ISG15*, *MKI67*, and *TOP2A* in six macrophage subclusters. (**G**) Dot plot showing expression of *CD163*, *ITGAM*, *CD27*, and *CCL5* in four different treatments of macrophages. (**H**) Chord diagram showing the ligand-receptor pair CXCL10-CXCR3 involved in interactions between three NK subclusters (particularly the *GZMA*^high^ NK cells) and M1-like macrophages (*IL1B*^+^ Mø, *IL6*^+^ Mø, *CCL5*^+^ Mø, and *IFITM3*^+^ Mø) as well as *S100A4*^+^ and *NEAT1*^+^ tumor cells, and the ligand-receptor pair XCL1/XCL2-XCR1 was inferred between *GZMA*^high^ NK cells and DCs and between *XCL1*^high^ NK cells and DCs

The presence of myeloid cells was confirmed using specific marker genes, including those for macrophages (*C1QA*, *C1QB*, and *APOE*), monocytes (*FCN1*, *VCAN*, and *S100A8*), classical DCs (*CD1C*, *XCR1*, and *CLEC9A*), and plasmacytoid DCs (pDCs, *LILRA4*, *CLEC4C*, and *LAMP5*) (Fig. 6A and Additional file 1: Fig. S13A). Neutrophils were not restored in the experimental process, which is consistent with other recent studies [62]. A small number of DCs and pDCs were found in the data. DCs expressed both CD11b (*ITGAE*) and CD103 (*ITGAM*), and *ITGAE* expression was higher than *ITGAM* in the DCs (Additional file 1: Fig. S13B), suggesting that DCs may be prone to the cDC2 phenotype. pDCs expressed high levels of *ICOSLG* (Additional file 1: Fig. S13B), which enables them to promote Treg expansion [63]. There was no DEGs in DCs between different drug groups, and there was no DEGs in pDCs between different drug groups, indicating that DCs and pDCs performed similar functions under different drug administration effects.

Six transcriptionally heterogeneous macrophage subclusters (*CCL5*^+^ Mø, *CXCL8*^+^ Mø, *IFITM3*^+^ Mø, *IL1B*^+^ Mø, *IL6*^+^ Mø, and *MMP12*^+^ Mø) were distinguished (Fig. 6B and Additional file 1: Fig. S13C). Typically, macrophages are separated into M1 and M2 subsets. We detected the expression of M1 (*e.g.*, *IL1A*, *IL1B*, *TNF*, and *IL12A*) and M2 genes (*e.g.*, *CD163*, *MARCO*, *MRC1*, and *CCL8*) [64, 65] in these six subclusters and calculated the M1 and M2 signature scores based on these marker genes (Fig. 6C and Additional file 1: Fig. S13D). *CXCL8*^+^ Mø and *MMP12*^+^ Mø cells displayed a higher M2 signature score than M1, whereas the remaining macrophage subclusters displayed higher M1 than M2 signature scores, indicating that *CXCL8*^+^ Mø and *MMP12*^+^ Mø cells may be more inclined to present an M2 signature while the other subclusters were more inclined to present an M1 signature. The SMI group exhibited the highest proportion of M1-like macrophages, followed by the PD-1+SMI, PD-1, and IgG groups, respectively (Fig. 6D).

The biological functions of the six macrophage groups were determined using Metascape analysis based on their DEGs (Fig. 6E). *CXCL8*^+^ Mø cells, which expressed high levels of *VEGFA* (Fig. 6F), were most abundant in the IgG and PD-1 groups. Positive regulation of angiogenesis and the response to hypoxia were enriched in *CXCL8*^+^ Mø cells (Fig. 6E), revealing that *CXCL8*^+^ Mø may induce angiogenesis in NSCLC. Matrix metalloproteinase (MMP)-9, MMP12, and β2 integrin ITGB2, which are associated with ECM degradation and cell invasion, were highly expressed in *MMP12*^+^ Mø cells (Fig. 6F). *MMP12*^+^ Mø cells were more abundant in the IgG and PD-1 groups than in the SMI and PD-1+SMI groups. Collagen catabolic process and NABA ECM regulators were enriched in *MMP12*^+^ Mø cells (Fig. 6E), suggesting that *MMP12*^+^ Mø may exert an ECM degradation function. *IFITM3*^+^ Mø cells were the dominant macrophage type in the PD-1+SMI group. The IFN-stimulated genes, *IFITM3* and *ISG15*, were highly expressed in *IFITM3*^+^ Mø cells (Fig. 6F). The response to IFNγ and the IGF2BP1 complex was enriched in *IFITM3*^+^ Mø cells (Fig. 6E), IFN-γ mediates an M1-like state in macrophages, suggesting that *IFITM3*^+^ Mø cells exert high pro-inflammatory effects. *MKI67* and *TOP2A* were highly expressed in *IL1B*^+^ Mø cells (Fig. 6F), in which G2/M transition and positive regulation of the cell cycle were enriched (Fig. 6E), suggesting that *IL1B*^+^ Mø cells may exert proliferative functions. *CCL5*^+^ Mø and *IL6*^+^ Mø cells were most abundant in the SMI group. Regulation of leukocyte activation and the IL-18 signaling pathway was enriched in *CCL5*^+^ Mø cells, and the cellular response to IL-1 and inflammatory response were enriched in *IL6*^+^ Mø cells (Fig. 6E), suggesting that these two subclusters are prone to the M1 state. Moreover, expression of *CD163*, *ITGAM*, *CCL5*, and *CD27* was lower in the macrophages of the PD-1+SMI group compared with that in the IgG, PD-1, and SMI groups (Fig. 6G), and higher expression of these genes in tumor-associated macrophages is associated with lower PFS and OS in patients with NSCLC and resistance to immunotherapy [66]. Myeloid-derived suppressor cells (MDSCs) include a population of monocytic MDSCs (M-MDSCs), granulocytic or polymorphonuclear MDSCs, and immature or early-stage MDSCs in cancer that suppress the immune microenvironment [67]. M-MDSCs are identified as CD33^+^CD11b^+^CD14^+^HLA-DR^−/low^ and resemble monocytes/macrophages in humans [67]. Here, monocytes, macrophages, and DCs were reevaluated using these marker genes. As shown in Fig. 6E and Additional file 1: Fig. S13E, *CXCL8*^+^ Mø cells were identified as M-MDSCs with functions related to angiogenesis and the hypoxia response, consistent with the immune-suppressive functions of MDSCs [67].

Moreover, we inferred three NK subclusters, particularly, the *GZMA*^high^ NK cells, may receive strong signals from M1-like macrophages (*IL1B*^+^ Mø, *IL6*^+^ Mø, *CCL5*^+^ Mø, and *IFITM3*^+^ Mø) as well as *S100A4*^+^ and *NEAT1*^+^ tumor cells via CXCL10-CXCR3 (Fig. 6H), which is essential for the recruitment of NK cells into the TME [57]. Due to the greater total proportion of *GZMA*^high^ and *XCL1*^high^ NK cells in the PD-1+SMI group compared with that in the PD-1 group (96.1% vs. 54.7%), the recruitment of NK cells and related antitumor effects may be greater in the PD-1+SMI group, which was consistent with phenotypic data. Second, NK cell recruitment of DCs into the TME and further activation of T cells to mediate antitumor effects were also identified. Intense interactions between *GZMA*^high^ NK cells and DCs and between *XCL1*^high^ NK cells and DCs via XCL1/XCL2-XCR1 and FLT3LG-FLT3 (Fig. 6H and Additional file 1: Fig. S13F) pairs were detected, suggesting that *GZMA*^high^ and *XCL1*^high^ NK cells may recruit and activate DCs into the tumor site. Additionally, we determined that *CD69*^+^ CD4^+^ T_RM_, *KLRB1*^+^ CD4^+^ Th17-like T, and *SELL*^+^ CD4^+^ T_N_ cells receive activation signals from DCs via CD40-CD40L (Additional file 1: Fig. S13F), whereas other costimulatory signals, including CD137-CD137L, OX40-OX40L, GITR-GITRL, and CD70-CD27, were not identified between DCs and T cells. Thus, our results reveal the complex functions of *GZMA*^high^ and *XCL1*^high^ NK cells that exist in the LUSC TME. Moreover, *GZMA*^high^ and *XCL1*^high^ NK cells may be recruited by M1-like macrophages and some tumor cells, which in turn directly kill or recruit DCs to activate T cells to kill tumor cells.

### Combination therapy of SMI and PD-1 inhibitor decreases the proportion of cancer metabolism reprogramming-associated fibroblasts and angiogenesis-associated endothelial cells

**Fig. 7:**
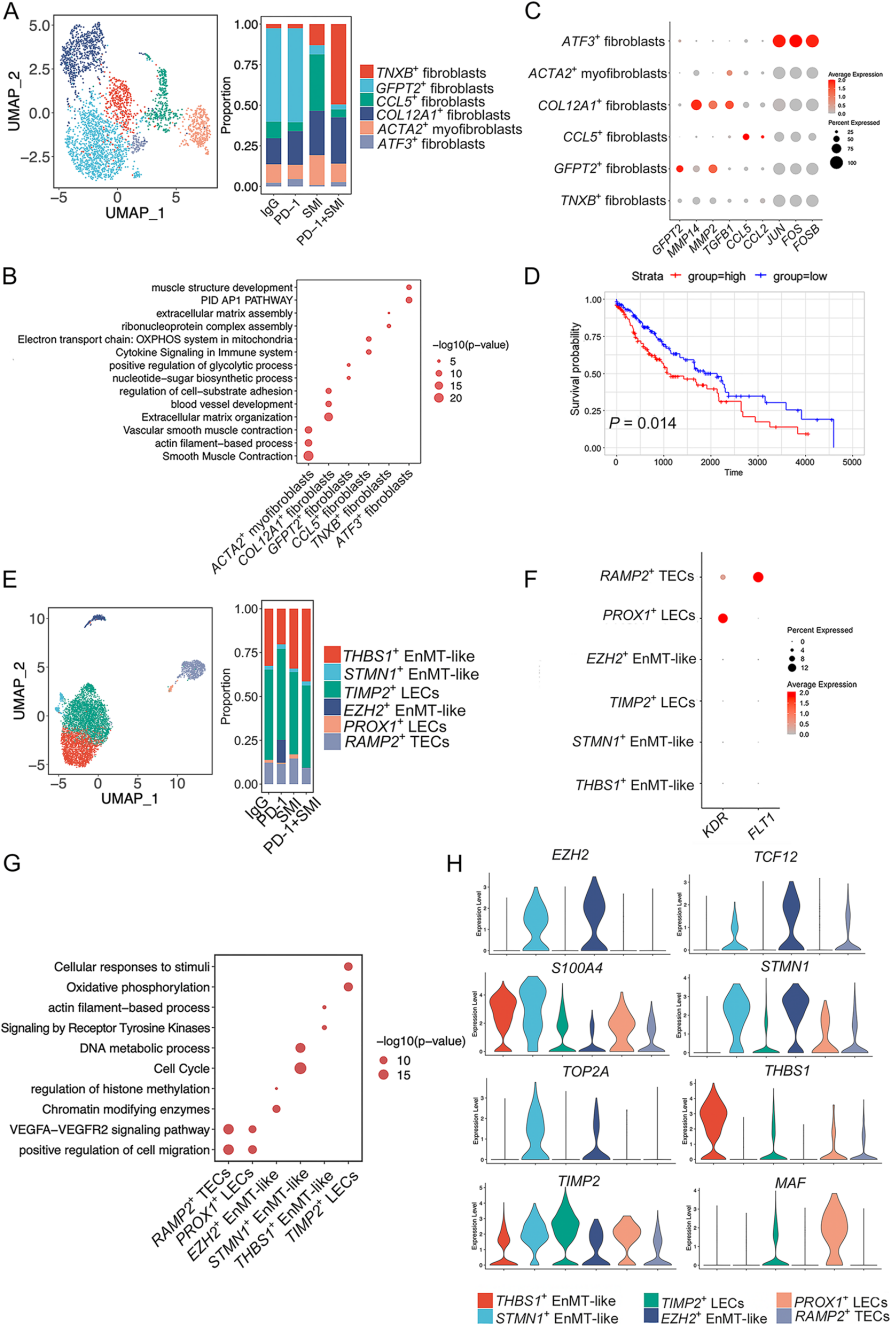
Transcriptional diversity in fibroblast and endothelial cells. (**A**) UMAP of five fibroblast subclusters. Relative proportion of these clusters in four different treatments were showed on the right. IgG: immunoglobulin G isotype control; PD-1: PD-1 immune-checkpoint blockade antibody; SMI: SMI monotherapy; PD-1+SMI: combination of anti-PD-1 and SMI. (**B**) Dot plot showing expression of *GFPT2*, *MMP14*, *MMP2*, *TGFB1*, *CCL5*, *CCL2*, *JUN*, *FOS*, and *FOSB* in five fibroblast subclusters. (**C**) Dot plot showing the top representative biological pathways of five fibroblast subclusters based on DEGs using Metascape analysis. (**D**) High level of *GFPT2* predicted poor prognosis in TCGA-LUSC.htseq_counts.tsv (*n* = 550 samples). Log-rank *P* < 0.05 was considered as statistically significant. (**E**) UMAP of six endothelial cell subclusters. Relative proportion of these clusters in four different treatments were showed on the right. (**F**) Dot plot showing expression of *KDR* and *FLT1* in six endothelial cell subclusters. (**G**) Dot plot showing the top representative biological pathways of six endothelial cell subclusters based on DEGs using Metascape analysis. (**H**) Violin plot showing expression of *EZH2*, *TCF12*, *S100A4*, *STMN1*, *TOP2A*, *THBS1*, *TIMP2*, and *MAF* in six endothelial cell subclusters

Cancer-associated fibroblasts (CAFs) contribute to tumor invasion, metabolism, immune suppression, and metastasis in NSCLC. Here, the fibroblasts were clustered into six subgroups after unsupervised cell clustering (Fig. 7A and Additional file 1: Fig. S14A). Next, we conducted Metascape analysis to explore the functions of these fibroblast subclusters based on their upregulated DEGs (Fig. 7B). A subgroup with high expression of *GFPT2* (Fig. 7C) was identified as the CAFs, which promote metabolism reprogramming by activating the hexosamine biosynthesis pathway in NSCLC [68]. This subgroup was the dominant type in the IgG and PD-1 groups. Metascape analysis revealed that positive regulation of the glycolytic and nucleotide-sugar biosynthetic processes was enriched in *GFPT2*^+^ fibroblasts (Fig. 7B). This suggests that *GFPT2*^+^ fibroblasts may be involved in cancer metabolism reprogramming. Patients with LUSC (TCGA-LUSC.htseq_counts.tsv) showing high expression of *GFPT2* exhibited lower overall survival (*P* < 0.05) than those with low expression (Fig. 7D), suggesting that *GFPT2*^+^ fibroblasts may be associated with poor survival in patients with NSCLC. Another subcluster of *CCL5*^+^ fibroblasts, which was abundant in the SMI group, also highly secreted *CCL2* (Fig. 7C). Both the CCL5 and CCL2 chemokines recruit MDSCs to the tumor site, and the CAF-MDSC axis affects ROS levels [68, 69]. Cytokine signaling in the immune system and the electron transport chain: OXPHOS system in mitochondria were enriched in the *CCL5*^+^ fibroblasts (Fig. 7B), suggesting that these fibroblasts may generate an immune-suppressive TME. *COL12A1*^+^ fibroblasts highly expressed *MMP2*, *MMP14*, and *TGFB1* (Fig. 7C), which are related to tumor cell invasion and ECM degradation. Simultaneously, ECM organization, blood vessel development, and regulation of cell-substrate adhesion were enriched in the *COL12A1*^+^ fibroblasts (Fig. 7B), suggesting that they may contribute to tumor invasion. Compared with the PD-1 group, the proportion of this subgroup was slightly increased in the combined group (Fig. 7A). The ribonucleoprotein complex and ECM assembly-associated subcluster *TNXB*^+^ fibroblasts were primarily found in the PD-1+SMI group (Fig. 7A-B). *TNXB* is associated with decreased MMP levels and downregulated during tumor progression. Furthermore, high expression of *TNXB* is correlated with a higher cancer survival prognosis [70], which showed that the *TNXB*^+^ fibroblasts may be associated with NSCLC cancer survival prognosis, further indicating that SMI assisting PD-1 inhibitor may prolong the survival of NSCLC patients by increasing the proportion of *TNXB*^+^ fibroblasts. Moreover, the AP-1 pathway associated genes, *JUN*, *FOS*, and *FOSB,* were highly expressed in *ATF3*^+^ fibroblasts (Fig. 7C), which are associated with senescence [71]. There had no DEGs in *ATF3*^+^ fibroblasts between different drug groups, indicating that this subpopulation performed similar functions under different drug administration. Lastly, smooth muscle contraction, actin filament-based processes, and vascular smooth muscle contraction were enriched in *ACTA2*^+^ myofibroblasts (Fig. 7B), which stimulate tumor progression via contraction of the ECM and promotion of EMT [72]. Metascape analysis was performed on the DEGs of *ACTA2*^+^ myofibroblasts from the PD-1+SMI group, and it was found that the regulation of intrinsic apoptosis signaling pathways was enriched (Additional file 1: Fig. S14B), indicating that the *ACTA2*^+^ myofibroblasts in the PD-1+SMI group may be in a state of apoptosis, and SMI assisting PD-1 inhibitor may inhibit the effect of the *ACTA2*^+^ myofibroblasts on tumor progression.

Endothelial cells are also major components of the stromal microenvironment. In the present study, 5,440 endothelial cells were re-clustered into six subclusters after unsupervised cell clustering (Fig. 7E and Additional file 1: Fig. S14C). *RAMP2*^+^ tumor endothelial cells (TECs) and *PROX1*^+^ lymphatic endothelial cells (LECs), were reduced in the PD-1+SMI group and highly expressed *VEGF*-associated receptor gene *KDR* (VEGFR1) and *FLT1* (VEGFR2) compared with other endothelial subclusters (Fig. 7F). Metascape analysis demonstrated that both subclusters were enriched for positive regulation of cell migration and the VEGFA-VEGFR2 signaling pathway (Fig. 7G), indicating that both subclusters were involved in angiogenesis and cell migration. Another subcluster that highly expressed the histone methyltransferase *EZH2* and its TF *TCF12* (Fig. 7H), which repress the vascular endothelial cadherin gene and facilitate endothelial-to-mesenchymal transition (EndMT) [73], was only found in the PD-1 group. Moreover, chromatin-modifying enzymes and regulation of histone methylation were enriched in *EZH2*^+^ EnMT-like cells (Fig. 7G), suggesting that *EZH2*^+^ cells are related to the EndMT process and epigenetic regulation. The other two subclusters *STMN1*^+^ EndMT-like cells and *THBS1*^+^ EndMT-like cells both expressed the mesenchymal biomarker *S100A4* [73], and one expressed high levels of the cell cycle genes *STMN1* and *TOP2A*, while the other expressed *THBS1* (Fig. 7H). The cell cycle and DNA metabolic processes were enriched in *STMN1*^+^ EndMT-like cells, while signaling by receptor tyrosine kinases and actin filament-based processes were enriched in *THBS1*^+^ EndMT-like cells (Fig. 7G). Compared with the IgG group, the proportion of the *THBS1*^+^ EndMT-like cells decreased in the PD-1 group and increased in the SMI group and PD-1+SMI group, with the highest proportion detected in the PD1+SMI group (Fig. 7E). *THBS1* inhibits tumor growth, cell migration, and neovascularization, and low expression of this gene is related to poor prognoses in NSCLC [74], which indicated that *THBS1*^+^ EndMT-like cells may be related to the prognosis of NSCLC, and SMI assisting PD-1 inhibitor may improve the prognosis of NSCLC by increasing the proportion of *THBS1*^+^ EndMT-like cells. The last subcluster expressed the inhibitor of metalloproteinase *TIMP2* and TF of lymphatic endothelial cells *MAF* (Fig. 7H) [75], and the proportion of *TIMP2*^+^ LECs was similar in different drug groups (Fig. 7E). Oxidative phosphorylation and cellular responses to stimuli were enriched in *TIMP2*^+^ LECs (Fig. 7G), and a previous study demonstrated that induction of oxidative phosphorylation inhibits pathological angiogenesis [76], suggesting that *TIMP2*^+^ LECs may suppress angiogenesis.

## Discussion

ICIs have revolutionized clinical treatments for cancer and are commonly applied in NSCLC treatment. Despite the promise of ICIs therapy, less than 20% of patients with NSCLC respond to immunotherapy alone, and a large proportion do not respond to PD-1/PD-L1 monotherapy, while some patients suffer severe irAEs. To achieve optimal therapeutic effects and reduce adverse events, a combination strategy is recommended. Owing to the multi-target and bidirectional regulation of immunity, TCM has emerged as a promising candidate for combination therapy with ICIs. In this study, we demonstrated that combination therapy of a PD-1 inhibitor and SMI significantly improved antitumor efficacy and prolonged survival without increasing irAEs in the LLC mouse and NCI-H226 HuNCG mouse models, providing preclinical evidence for a new combination therapy strategy for NSCLC. Moreover, the main effect of SMI in the combination therapy was identified as increased tumor-infiltrating *GZMA*^high^ and *XCL1*^high^ NK subclusters with cytotoxic and chemokine signatures. Given the essential role of NK cells in the antitumor immune response via direct mediation of tumor lysis and recruitment of other antitumor immune cells, our data provides a potential direction for subsequent screening of ICIs combination strategies.

Our findings revealed that the proportion of tumor-infiltrating NK cells was significantly increased following combination therapy in both the LUAD and LUSC humanized mouse models compared with that in the monotherapy, suggesting that the combination of PD-1 inhibitor with SMI recruited NK cells into the TME of NSCLC. NK cells perform distinct functional roles in human melanoma as both the cytotoxic subpopulation against malignant cells and subpopulation that shapes the DC response to tumors [55, 57]. Consistently, the tumor-infiltrating *GZMA*^high^ NK subcluster expressing a high level of cytotoxic genes (*GZMA*, *GZMB*, *GZMK*, *GZMM*, *GZMH*, *PRF1*, and *GNLY*) and FASL and TRAIL genes was most enriched in the PD-1+SMI group. Another tumor-infiltrating *XCL1*^high^ NK subcluster that expressed a high level of genes associated with the recruitment of DCs (*XCL1*, *XCL2*, and *FLT3LG*) was more highly detected in the PD-1+SMI than in the PD-1 group. Besides, higher levels of *GZMA* and *XCL1* were observed in the NK cells from the PD-1+SMI group compared with those in the remaining groups, suggesting that the PD-1 inhibitor combined with SMI induced cytotoxic NK cells to infiltrate the NSCLC tumor and may recruit DCs into the tumor via NK cells, thus improving the antitumor efficacy. The present study found that the amount of GzmA secreted by NK cells was significantly improved following combination therapy compared with that in the PD-1 inhibitor monotherapy. Furthermore, the proportion of exhausted NKT, *CXCL13*^+^ CD8^+^ T_EX_, *ENTPD1*^+^ CD8^+^ T_EX_, and *PDCD1*^+^ CD4^+^ T_EX_ cells, which highly express the inhibitory receptors *PDCD1*, *TIGIT*, *LAG3*, *HAVCR2*, and *CTLA4*, was notably decreased in the PD-1+SMI group compared with that in the remaining groups. Lastly, the cytotoxic gene signature score was higher while the exhausted gene signature score was lower in all T and NK cells in the PD-1+SMI group than in the remaining groups. This suggests that the PD-1 inhibitor combined with SMI blocks inhibitory receptors on NK and T cells and restores their antitumoral activity in NSCLC more effectively than PD-1 inhibitor monotherapy. Recently, Hsu et al. [77] proposed that NK cells would mediate the effect of PD-1/PD-L1 antibody therapy along with T cells. Wan et al. [78] found bispecific PD-1 and PD-L1 inhibitor not only induced T cells but also NK cells to be more active and cytotoxic in high-grade serous ovarian cancer, while neither monospecific antibody induced NK cell activation. Since SMI had been reported to increase activity of NK cells combined with chemotherapy [79], combination of PD-1 inhibitor and SMI may generate synergistic therapeutic effect to recruit and activate NK cell in NSCLC.

In addition, we demonstrated complex cell–cell communication between NK cell, tumor cell, T cell, DC, and macrophage components. We inferred highly significant interactions between *GZMA*^high^ NK cells and tumor subclusters and between *XCL1*^high^ NK cells and tumor subclusters involved in the activation of death receptor systems (TRAIL-TNFRS10B and FASL-FAS) in tumor cells. These interactions may induce tumor cells apoptosis. M1-like macrophages and some tumor cells recruited NK cells via CXCL10-CXCR3. Additionally, *GZMA*^high^ and *XCL1*^high^ NK cells recruited and activated DCs via XCL1/XCL2-XCR1 and FLT3LG-FLT3, which activate *CD69*^+^ CD4^+^ T_RM_, *KLRB1*^+^ CD4^+^ Th17-like T, and *SELL*^+^ CD4^+^ T_N_ cells via CD40-CD40L and recruit T cells via CXCL10-CXCR3, thus promoting antitumor efficacy. Recently, agonist anti-CD40 antibody and CD40L alone or in combination with other therapies have been shown to significantly reduce the growth of B cell lymphomas, breast and ovarian carcinomas, and several other solid tumors and prolong overall survival of patients [80]. Moreover, *CXCL10* suppresses myeloma by recruiting effector CD8^+^ T, CD4^+^ T, and NK cells to the tumor site, and combining *CXCL10* with radiotherapy enhances its therapeutic efficacy in cervical cancer [81]. The PD-1 inhibitor and SMI combination induced a higher proportion of *GZMA*^high^ NK cells, *XCL1*^high^ NK cells, M1-like macrophages, *CD69*^+^ CD4^+^ T_RM_ cells, *KLRB1*^+^ CD4^+^ Th17-like T cells, and *SELL*^+^ CD4^+^ T_N_ cells compared with that in PD-1 inhibitor monotherapy. Therefore, combination of PD-1 inhibitor and SMI enhanced antitumor efficacy mainly by recruiting more NK cells into tumor site and activating the cytotoxicity function of NK cells. The activated NK cells would in turn to enlist and trigger DCs and T cells.

High heterogeneity of carcinoma cells was detected following administration of different treatments. Notably, malignant cells from the PD-1+SMI group were mainly found in the apoptotic branch (*NNMT*^+^ and *FSTL1*^+^ tumor cells), whereas those from the PD-1 and IgG groups were clustered in the invasive and migrative branches (*H3F3B*^+^ and *FOSB*^+^ tumor cells), and finally, malignant cells from the SMI group were mainly clustered in the CSCs and EMT branches (*NEAT1*^+^ and *S100A4*^+^ tumor cells). This phenomenon may be associated with greater cytotoxic NK cell infiltration into the tumor in the PD-1+SMI group than in the remaining groups, indicating that the PD-1 inhibitor combined with SMI may enhance the antitumor efficacy via NK cell-mediated tumor cell apoptosis. High expression of *ERH*, one of the *NNMT*^+^ tumor cell-associated genes, predicted longer survival in LUSC. *ERH* plays opposing roles in different tissue-specific cancers. Overexpression of *ERH* in gastric cancer decreases cell migration and invasion and is associated with a good prognosis, while in ovarian cancer, *ERH* promotes metastasis and invasion by regulating EMT [82]. High expression of *FOSB*^+^, *NEAT1*^+^, or *XIST*^+^ tumor cell-associated genes such as *FSTL3*, *VTN*, *PAPPA*, *CCN1*, *RRAD*, and *GPRIN3* may predict poor survival in patients with LUSC, suggesting that these genes act as prognosis biomarkers.

This study also highlights the decrease in angiogenic features observed in the TME in the PD-1+SMI group compared with that in the PD-1 group. Specifically, the proportion of *RAMP2*^+^ TECs and *PROX1*^+^ LECs with high expression of VEGFR1 and VEGFR2, and *CXCL8*^+^ M-MDSCs with high expression of *VEGFA*, which induce angiogenesis, were decreased in the PD-1+SMI compared with that in the PD-1 group. VEGF induces vascular abnormalities and augments the immune suppressive activity of MDSCs, Tregs, and tumor-associated macrophages in lung cancer, colon cancer, and hepatocellular carcinoma, which in turn drives angiogenesis to suppress antitumor immunity [83]. Therefore, several combination therapies that focus on combining angiogenesis and PD-1/PD-L1 pathways or MDSCs and PD-L1 inhibitors have emerged [84, 85]. Our data suggests that the PD-1 inhibitor combined with SMI may reduce angiogenesis and MDSCs in NSCLC, with SMI potentially augmenting the PD-1 inhibitor. Furthermore, cancer metabolism reprogramming-associated *GFPT2*^+^ CAFs were reduced in the PD-1+SMI compared with that in the PD-1 group. *GFPT2* regulates cell migration and invasion in NSCLC[86], and its expression is positively correlated with poor survival in patients with serous ovarian cancer, colon adenocarcinoma, and LUAD [86–88]. For the first time, our study reveals that elevated *GFPT2* expression is correlated with poor clinical outcomes in LUSC, suggesting that the PD-1 inhibitor combined with SMI may prolong patient survival in NSCLC by decreasing the proportion of *GFPT2*^+^ CAFs. Thus, *GFPT2*^+^ CAFs may serve as potential target cells for the treatment of NSCLC.

However, the immunomodulatory effects of SMI in NSCLC are context dependent. Indeed, the effects of SMI in combination with PD-1 differ from those of SMI alone. For instance, malignant cells in the SMI group mainly displayed CSCs and EMT phenotypes, whereas malignant cells in the PD-1+SMI group mainly exhibited an apoptosis phenotype. The SMI group displayed higher levels of *XCL1*^high^ NK cells, whereas the PD-1+SMI group showed a higher level of *GZMA*^high^ NK cells, and exhausted T/NK cells were higher in the SMI than in the PD-1+SMI group. This finding was consistent with a recent study in which Zhong et al. [89] showed that combined treatment of SMI and 5-fluorouracil (5FU) significantly suppressed tumor growth (65.1%), whereas SMI monotherapy exerted a minimal effect (5.1%). Furthermore, tumor blood flow in the combination group was significantly increased compared with that in the control group while SMI monotherapy did not have an effect [89], demonstrating that the regulatory effects of SMI may depend on combination with other drugs, including 5FU.

## Conclusions

In summary, we provide preclinical evidence for a novel combination therapy comprising ICIs and TCM for anti-NSCLC treatment. Treatment with PD-1 inhibitor combined with the immunomodulator SMI improved antitumor efficacy and extended survival without increasing irAEs in NSCLC. Using scRNA-seq, we determined that the combination of PD-1 inhibitor and SMI induced cytotoxic NK cells to infiltrate the NSCLC tumor, recruited DCs to tumors via NK cell activation, alleviated the exhausted state of T and NK cells, reduced angiogenic characteristics in the TME, and diminished cancer metabolism reprogramming-associated CAFs. Our study suggests that NK cells may be used as a drug screening target for successful combination strategies with ICIs.

## Supplementary Information

**Additional file 1:** **Fig. S1** The hematoxylin-eosin (HE) staining and the proportion of tumor-infiltrating immune cells after different treatments in lewis lung carcinoma (LLC) mouse model. (A) Representative HE staining of liver of the different treatments. Black arrow means infiltration of inflammatory cells. IgG: immunoglobulin G isotype control; PD-1: programmed death-1 (PD-1) immune-checkpoint blockade antibody; SMI: shenmai injection (SMI) monotherapy; PD-1+SMI: combination of anti-PD-1 and SMI. (B) Representative HE staining of lung in the different treatments. (C) The proportion of tumor-infiltrating CD8^+^ T, CD4^+^ T, regulatory T cells (Tregs), and B cells in the different treatments. **Fig. S2** The biochemical indices of the different treatments in LLC mouse model. Indices of ATL, AST, *n =* 5; others, *n =* 7. IgG: immunoglobulin G isotype control; PD-1: PD-1 immune-checkpoint blockade antibody; SMI: SMI monotherapy; PD-1+SMI: combination of anti-PD-1 and SMI. **Fig. S3** Representative HE staining of heart, kidney, and spleen of the different treatments in LLC mouse model. IgG: immunoglobulin G isotype control; PD-1: PD-1 immune-checkpoint blockade antibody; SMI: SMI monotherapy; PD-1+SMI: combination of anti-PD-1 and SMI. **Fig. S4 **Photographs of all tumors after the different treatments in the lung squamous cell carcinoma humanized mouse model. IgG: immunoglobulin G isotype control; PD-1: PD-1 immune-checkpoint blockade antibody; SMI: SMI monotherapy; PD-1+SMI: combination of anti-PD-1 and SMI. IgG, SMI, and PD-1+SMI groups, *n =* 7; PD-1 group, *n =* 6. **Fig. S5** Representative HE staining of liver of the different treatments in humanized mouse model. IgG: immunoglobulin G isotype control; PD-1: PD-1 immune-checkpoint blockade antibody; SMI: SMI monotherapy; PD-1+SMI: combination of anti-PD-1 and SMI. **Fig. S6** Representative HE staining of lung of the different treatments in humanized mouse model. IgG: immunoglobulin G isotype control; PD-1: PD-1 immune-checkpoint blockade antibody; SMI: SMI monotherapy; PD-1+SMI: combination of anti-PD-1 and SMI. **Fig. S7** The biochemical indices of the different treatments in the humanized mouse model. IgG: immunoglobulin G isotype control; PD-1: PD-1 immune-checkpoint blockade antibody; SMI: SMI monotherapy; PD-1+SMI: combination of anti-PD-1 and SMI. IgG, SMI, and PD-1+SMI groups, *n =* 7; PD-1 group, *n =* 6. **Fig. S8** Single-cell transcriptome profiles of different treatments in the humanized mouse model. (A) The expression of marker genes for eight cell types. (B) Uniform manifold approximation and projection (UMAP) projection within each sample origin. IgG: immunoglobulin G isotype control; PD-1: PD-1 immune-checkpoint blockade antibody; SMI: SMI monotherapy; PD-1+SMI: combination of anti-PD-1 and SMI. **Fig. S9** Seven subclusters of malignant epithelial cells were identified upon different treatments. (A) Dot plot showing the expression of *KRT8*, *KRT18*, and *KRT19* in eight cell types. (B) Heatmap of differentially expressed genes (DEGs) among seven malignant epithelial subclusters. *NEAT1*^+^: *NEAT1*^+^ tumor cells; *FOSB*^+^: *FOSB*^+^ tumor cells; *NNMT*^+^: *NNMT*^+^ tumor cells; *S100A4*^+^: *S100A4*^+^ tumor cells; *XIST*^+^: *XIST*^+^ tumor cells; *FSTL1*^+^: *FSTL1*^+^ tumor cells; *H3F3B*^+^: *H3F3B*^+^ tumor cells. (C) Heatmap showing the expression of cancer stem cells (CSCs) genes in seven malignant epithelial subclusters. (D) Dot plot showing the expression of *CD44*, *ALCAM*, and *FSTL1* in seven malignant epithelial subclusters. (E) Violin plot showing the *FSTL1*^+^ tumor cells had higher CSCs score compared with that in other subclusters except for *NEAT1*^+^ and *XIST*^+^ tumor cells. **Fig. S10 **Transcriptome heterogeneous and dynamics in seven malignant epithelial subclusters. (A) Top 10 biological pathways of seven malignant epithelial clusters based on DEGs using Metascape analysis. *NEAT1*^+^: *NEAT1*^+^ tumor cells; *FOSB*^+^: *FOSB*^+^ tumor cells; *NNMT*^+^: *NNMT*^+^ tumor cells; *S100A4*^+^: *S100A4*^+^ tumor cells; *XIST*^+^: *XIST*^+^ tumor cells; *FSTL1*^+^: *FSTL1*^+^ tumor cells; *H3F3B*^+^: *H3F3B*^+^ tumor cells. (B) Unsupervised transcriptional trajectory of seven malignant epithelial subsets predicted by Monocle 3. (C) High level of *FOSB*^+^ tumor cell-associated genes (*FSTL3* and *VTN*), *NEAT1*^+^ tumor cell-associated genes (*PAPPA*, *CCN1*, and *RRAD*), and *XIST*^+^ tumor cell-associated genes (*GPRIN3*) predicted poor prognosis in TCGA-LUSC.htseq_counts.tsv (*n* = 550 samples), whereas high level of *NNMT*^+^ tumor cell-associated genes (*ERH*) exhibited higher overall survival. Log-rank *P <* 0.05 was considered as statistically significant. **Fig. S11** The top 10 Transcription factors (TFs) in each malignant epithelial subcluster were inferred using SCENIC analysis. *NEAT1*^+^: *NEAT1*^+^ tumor cells; *FOSB*^+^: *FOSB*^+^ tumor cells; *NNMT*^+^: *NNMT*^+^ tumor cells; *S100A4*^+^: *S100A4*^+^ tumor cells; *XIST*^+^: *XIST*^+^ tumor cells; *FSTL1*^+^: *FSTL1*^+^ tumor cells; *H3F3B*^+^: *H3F3B*^+^ tumor cells. **Fig. S12** Single-cell transcriptome profiles of NK and T cells. (A) Heatmap of DEGs among four NK cell subclusters. (B) Dot plot showing expression of *PDCD1*, *TIGIT*, *CTLA4*, *LAG3*, and *HAVCR2* in four NK clusters. (C) Dot plot showing expression of *FASLG*, *TNFSF10*, and *FLT3LG* in *GZMA*^high^ NK, *HLA-DRB1*^high^ NK, *XCL1*^high^ NK clusters. (D) Dot plot showing expression of *GZMA* and *CMC1* in four different treatments. IgG: immunoglobulin G isotype control; PD-1: PD-1 immune-checkpoint blockade antibody; SMI: SMI monotherapy; PD-1+SMI: combination of anti-PD-1 and SMI. (E) Heatmap of T cell respective markers among thirteen T cell subclusters. (F) Dot plot showing expression of *FOXP3*, *IL2RA*, and *IKZF2* in four different treatments of Tregs. (G) Heatmap showing express of *CCR6* and *RORC* among thirteen T cell subclusters. (H) Top biological pathways of *KLRB1*^+^ CD4^+^ T helper 17-like T cells (*KLRB1*^+^ Th17-like CD4^+^ T cells) based on DEGs using Metascape analysis. (I) Violin plot showing the mean score of the cytotoxic or exhausted signature across four different treatments in T and NK cells. ^***^P < 0.001, ^****^P < 0.0001. **Fig. S13** Single-cell transcriptome profiles of myeloid cells. (A) Heatmap of DEGs among macrophages, monocytes, classical DCs, and plasmacytoid DCs (pDCs). (B) Violin plot showing expression of *ITGAE*, *ITGAM*, and *ICOSLG* in classical DCs and pDCs. (C) Heatmap of DEGs among six macrophages subclusters. (D) Heatmap describing the expression of M1 and M2 feature genes in six macrophage subclusters. (E) Dot plot showing expression of *CD33*, *CD14*, *ITGAM*, *HLA-DRA*, and *HLA-DRB1* in myeloid subclusters. (F) Chord diagram showing the ligand-receptor pair FTL3-FTL3LG involved in *GZMA*^high^ NK cells and DCs and between *XCL1*^high^ NK cells and DCs, and the ligand-receptor pair CD40-CD40LG involved in DCs and *CD69*^+^ CD4^+^ T_RM_, *KLRB1*^+^ CD4^+^ Th17-like T, and *SELL*^+^ CD4^+^ T_N_ cells. **Fig. S14** Single-cell transcriptome profiles of fibroblast and endothelial cells. (A) Heatmap of DEGs among fibroblast sublcusters. (B) Top biological pathways of based on DEGs of *ACTA2*^+^ myofibroblasts from the PD-1+SMI group using Metascape analysis. (C) Heatmap of DEGs among endothelial sublcusters.

**Additional file 2:** **Table S1** The representative marker genes of eight cell types. **Table S2** Biological pathways analysis using Metascape analysis. **Table S3** Top ten TFs in the tumor subclusters. **Table S4** The representative marker genes of T and NK cell subclusters.

## Data Availability

The scRNA-seq data are available upon request by contact with the corresponding author. The bulk RNA-seq data of LUSC (dataset ID: TCGA-LUSC.htseq_counts.tsv) were downloaded from TCGA database.

## Abbreviations

ALT: Alanine aminotransferase
APC: Antigen-presenting cell
AST: Aspartate aminotransferase
CAFs: Cancer-associated fibroblasts
CCI: Cell-cell interaction
CNV: Copy number variation
CSCs: Cancer stem cells
DCs: Dendritic cells
DEGs: Differentially expressed genes
ECM: Extracellular matrix
ELISA: Enzyme-linked immunosorbent assay
EMT: Epithelial-mesenchymal transition
EndMT: Endothelial-to-mesenchymal transition
FASL: Fas ligand
HE: Hematoxylin-eosin
GO: Gene Ontology
GzmA: Granzyme A
HLA: Human leukocyte antigen
HPD: Hyperprogressive disease
HuNCG: Humanized NOD/ShiLtJGpt-*Prkdc*^*em26Cd52*^*Il2rg*^*em26Cd22*^/Gpt
ICIs: Immune checkpoint inhibitors
IFN-γ: Interferon-gamma
IL: Interleukin
irAEs: Immune-related adverse events
KEGG: Kyoto Encyclopedia of Genes and Genomes
LECs: Lymphatic endothelial cells
LLC: Lewis lung carcinoma
LUAD: Lung adenocarcinoma
LUSC: Lung squamous cell carcinoma
MDSC: Myeloid-derived suppressor cell
m-MDSC: Monocytic myeloid-derived suppressor cell
NCG: NOD/ShiLtJGpt-*Prkdc*^*em26Cd52*^*Il2rg*^*em26Cd22*^/Gpt
NK: Natural killer
NKT: Nature killer T
NSCLC: Non-small cell lung cancer
OS: Overall survival
PD-1: Programmed death-1
pDC: Plasmacytoid dendritic cell
PD-L1: Programmed death-ligand 1
PBS: Phosphate buffer saline
PFS: Progression-free survival
SCENIC: Single-cell regulatory network inference
scRNA-seq: Single-cell RNA sequencing
SMI: Shenmai injection
TCM: Traditional Chinese medicine
TCGA: The Cancer Genome Atlas
TECs: Tumor endothelial cells
TFs: Transcription factors
TGF-β: Transforming growth factor-beta
TME: Tumor microenvironment
TNF: Tumor necrosis factor
Tregs: Regulatory T cells
TRAIL: TNF-related apoptosis-inducing ligand
UMAP: Uniform manifold approximation and projection
VEGF: Vascular endothelial growth factor
VEGFR: Vascular endothelial growth factor receptor
