# Supplementary material for: Single-cell RNA sequencing reveals enhanced antitumor immunity after combined application of PD-1 inhibitor and Shenmai injection in non-small cell lung cancer
Source: Cell Commun Signal. 2023 Jul 10;21:169. doi: 10.1186/s12964-023-01184-3 (PMC10332015; doi:10.1186/s12964-023-01184-3)
Supplement: Supplementary file 2 — Additional file 1: Fig. S1 The hematoxylin-eosin (HE) staining and the proportion of tumor-infiltrating immune cells after different treatments in lewis lung carcinoma (LLC) mouse model. (A) Representative HE staining of liver of the different treatments. Black arrow means infiltration of inflammatory cells. IgG: immunoglobulin G isotype control; PD-1: programmed death-1 (PD-1) immune-checkpoint blockade antibody; SMI: shenmai injection (SMI) monotherapy; PD-1+SMI: combination of anti-PD-1 and SMI. (B) Representative HE staining of lung in the different treatments. (C) The proportion of tumor-infiltrating CD8+ T, CD4+ T, regulatory T cells (Tregs), and B cells in the different treatments. Fig. S2 The biochemical indices of the different treatments in LLC mouse model. Indices of ATL, AST, n = 5; others, n = 7. IgG: immunoglobulin G isotype control; PD-1: PD-1 immune-checkpoint blockade antibody; SMI: SMI monotherapy; PD-1+SMI: combination of anti-PD-1 and SMI. Fig. S3 Representative HE staining of heart, kidney, and spleen of the different treatments in LLC mouse model. IgG: immunoglobulin G isotype control; PD-1: PD-1 immune-checkpoint blockade antibody; SMI: SMI monotherapy; PD-1+SMI: combination of anti-PD-1 and SMI. Fig. S4 Photographs of all tumors after the different treatments in the lung squamous cell carcinoma humanized mouse model. IgG: immunoglobulin G isotype control; PD-1: PD-1 immune-checkpoint blockade antibody; SMI: SMI monotherapy; PD-1+SMI: combination of anti-PD-1 and SMI. IgG, SMI, and PD-1+SMI groups, n = 7; PD-1 group, n = 6. Fig. S5 Representative HE staining of liver of the different treatments in humanized mouse model. IgG: immunoglobulin G isotype control; PD-1: PD-1 immune-checkpoint blockade antibody; SMI: SMI monotherapy; PD-1+SMI: combination of anti-PD-1 and SMI. Fig. S6 Representative HE staining of lung of the different treatments in humanized mouse model. IgG: immunoglobulin G isotype control; PD-1: PD-1 immune-checkpoint blockade an [file 12964_2023_1184_MOESM1_ESM.docx]

**Additional file 1**


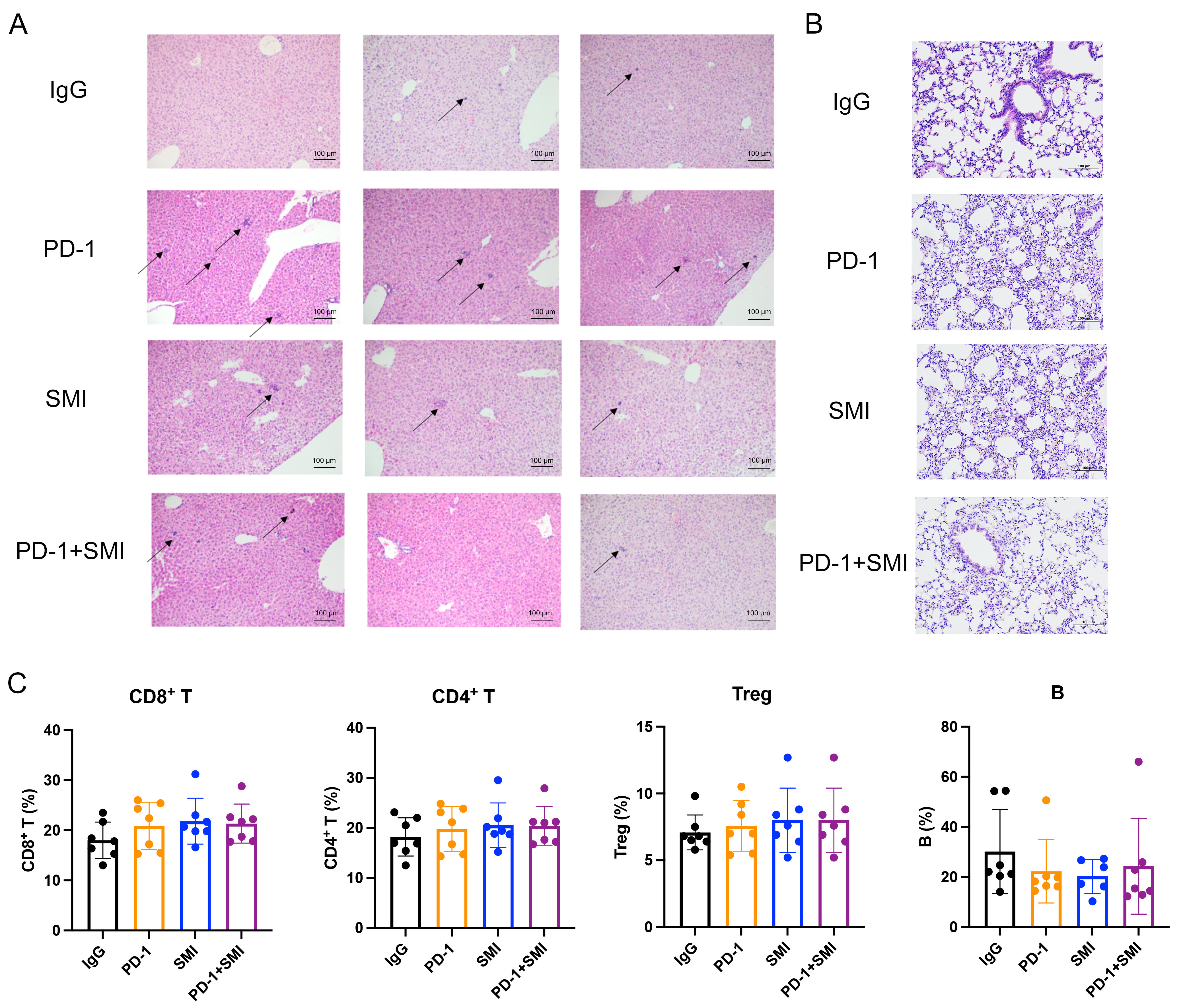


**Fig. S1** The hematoxylin-eosin (HE) staining and the proportion of tumor-infiltrating immune cells after different treatments in lewis lung carcinoma (LLC) mouse model. (A) Representative HE staining of liver of the different treatments. Black arrow means infiltration of inflammatory cells. IgG: immunoglobulin G isotype control; PD-1: programmed death-1 (PD-1) immune-checkpoint blockade antibody; SMI: shenmai injection (SMI) monotherapy; PD-1+SMI: combination of anti-PD-1 and SMI. (B) Representative HE staining of lung in the different treatments. (C) The proportion of tumor-infiltrating CD8^+^ T, CD4^+^ T, regulatory T cells (Tregs), and B cells in the different treatments.


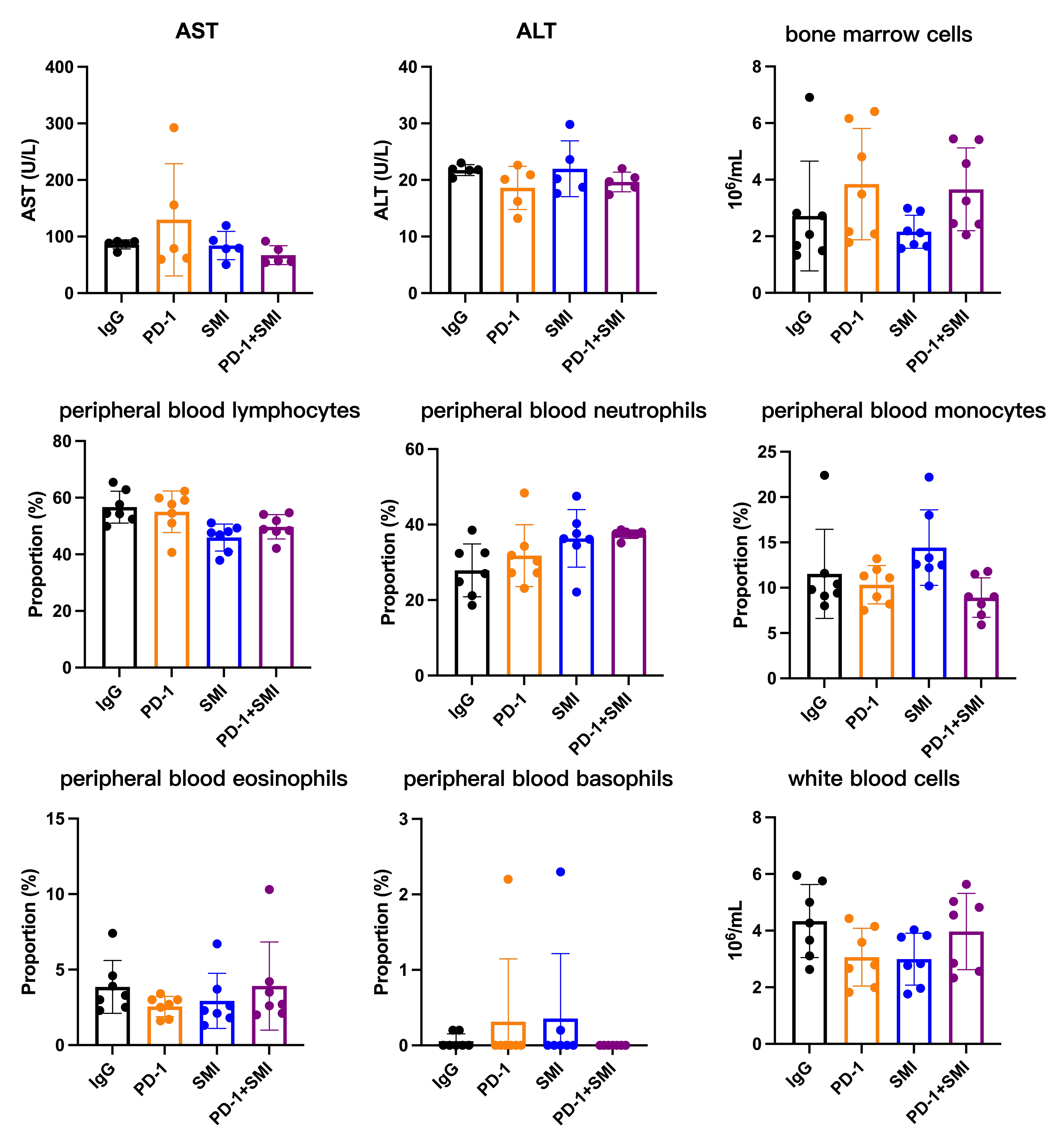


**Fig. S2** The biochemical indices of the different treatments in LLC mouse model. Indices of ATL, AST, n = 5; others, n = 7. IgG: immunoglobulin G isotype control; PD-1: PD-1 immune-checkpoint blockade antibody; SMI: SMI monotherapy; PD-1+SMI: combination of anti-PD-1 and SMI.


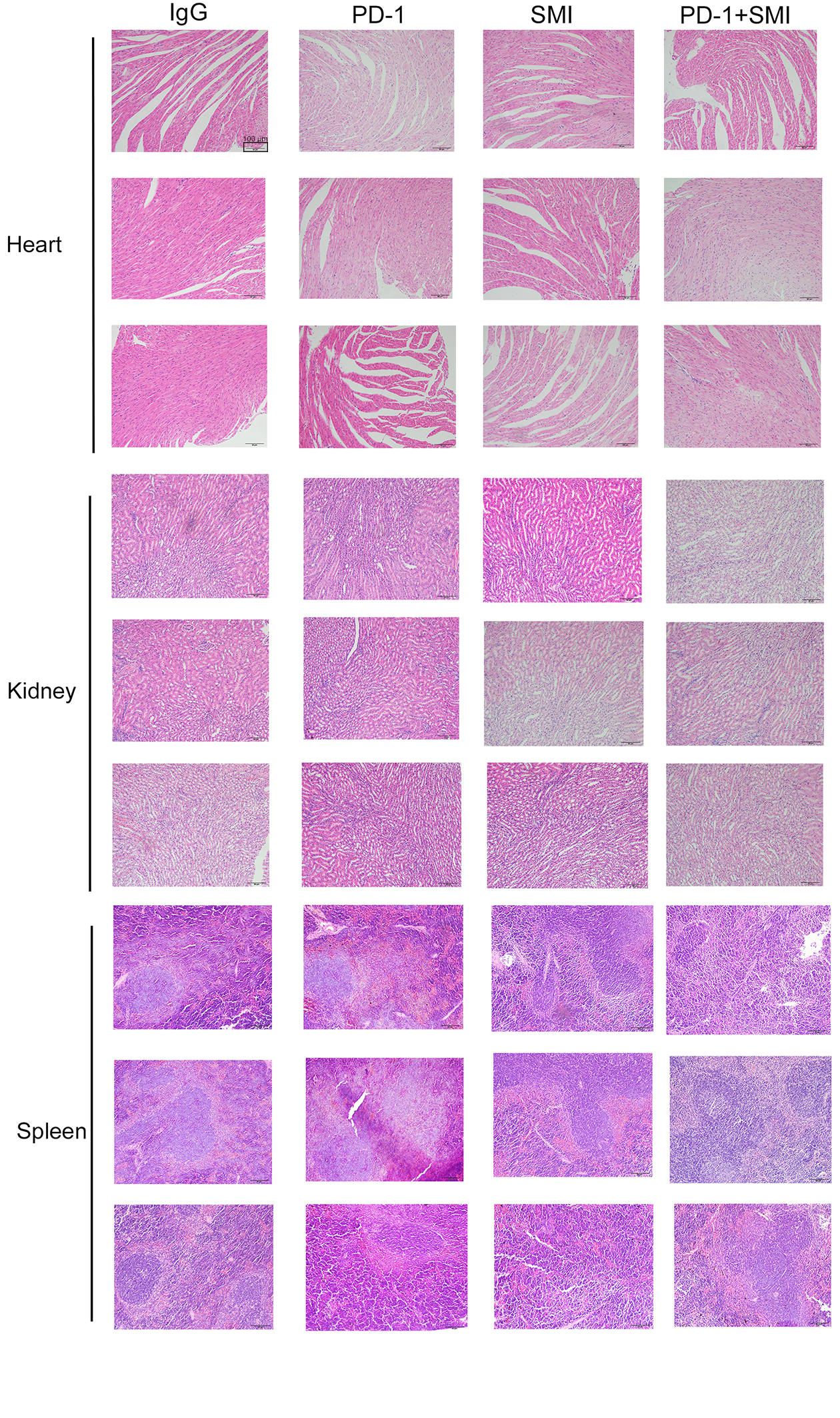


**Fig. S3** Representative HE staining of heart, kidney, and spleen of the different treatments in LLC mouse model. IgG: immunoglobulin G isotype control; PD-1: PD-1 immune-checkpoint blockade antibody; SMI: SMI monotherapy; PD-1+SMI: combination of anti-PD-1 and SMI.


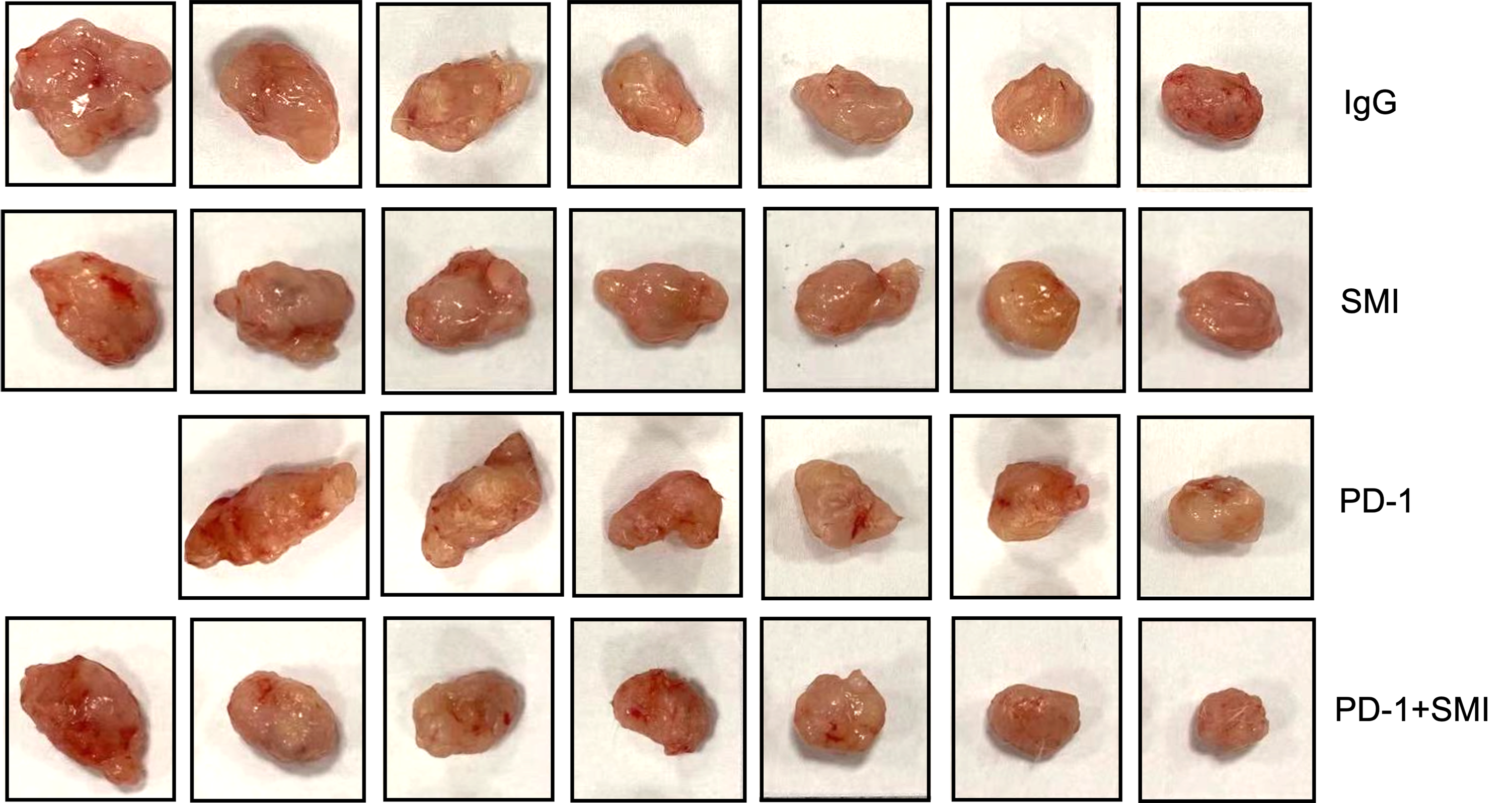


**Fig. S4** Photographs of all tumors after the different treatments in the lung squamous cell carcinoma humanized mouse model. IgG: immunoglobulin G isotype control; PD-1: PD-1 immune-checkpoint blockade antibody; SMI: SMI monotherapy; PD-1+SMI: combination of anti-PD-1 and SMI. IgG, SMI, and PD-1+SMI groups, n = 7; PD-1 group, n = 6.


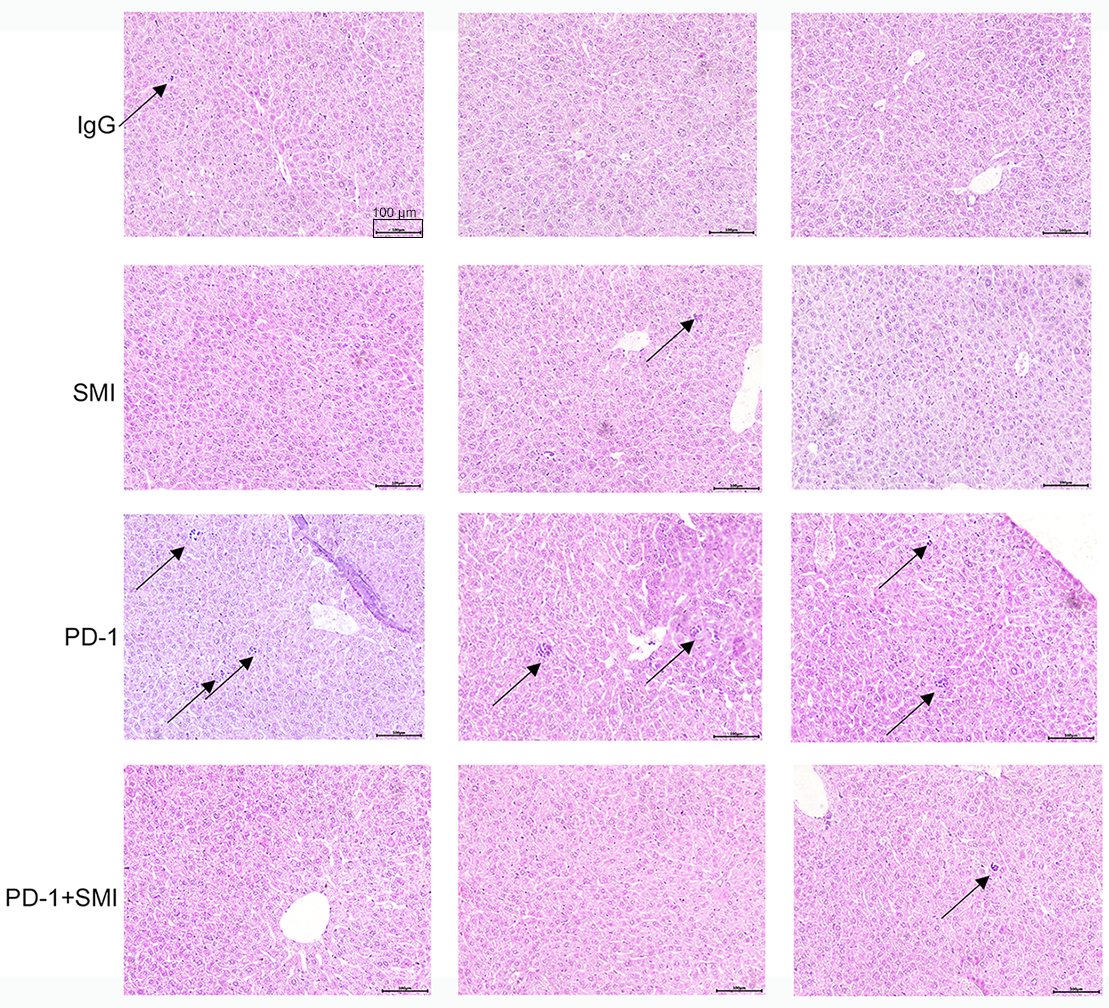


**Fig. S5** Representative HE staining of liver of the different treatments in humanized mouse model. IgG: immunoglobulin G isotype control; PD-1: PD-1 immune-checkpoint blockade antibody; SMI: SMI monotherapy; PD-1+SMI: combination of anti-PD-1 and SMI.


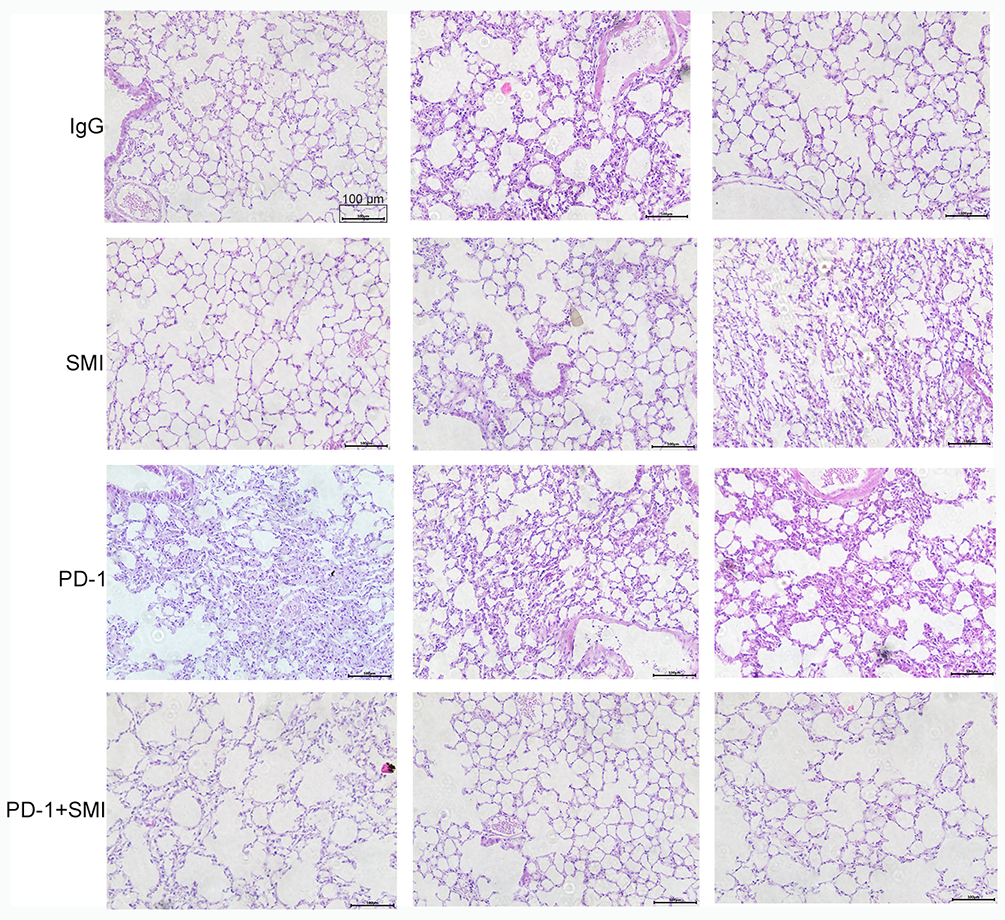


**Fig. S6** Representative HE staining of lung of the different treatments in humanized mouse model. IgG: immunoglobulin G isotype control; PD-1: PD-1 immune-checkpoint blockade antibody; SMI: SMI monotherapy; PD-1+SMI: combination of anti-PD-1 and SMI.


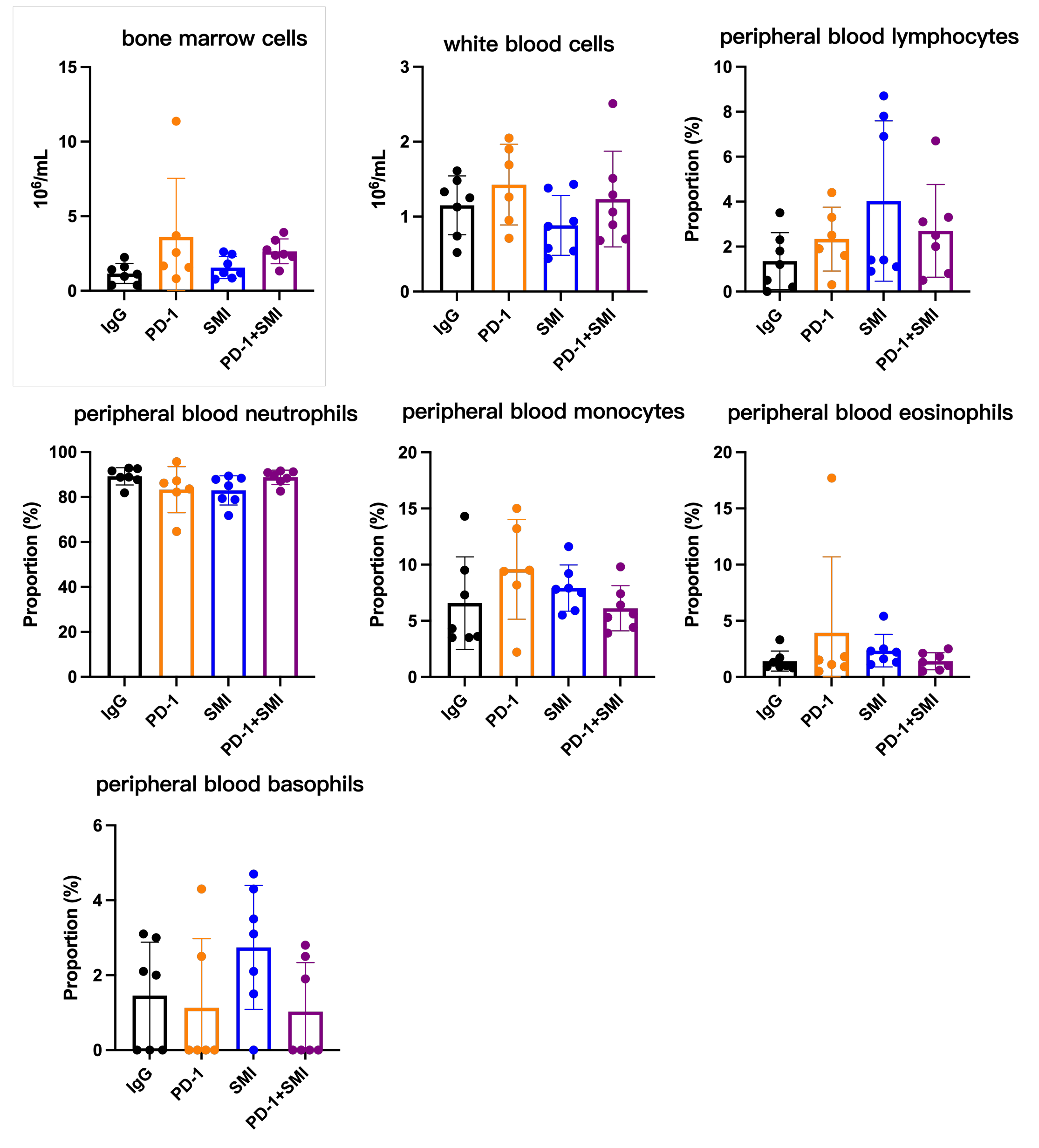


**Fig. S7** The biochemical indices of the different treatments in the humanized mouse model. IgG: immunoglobulin G isotype control; PD-1: PD-1 immune-checkpoint blockade antibody; SMI: SMI monotherapy; PD-1+SMI: combination of anti-PD-1 and SMI. IgG, SMI, and PD-1+SMI groups, n = 7; PD-1 group, n = 6.


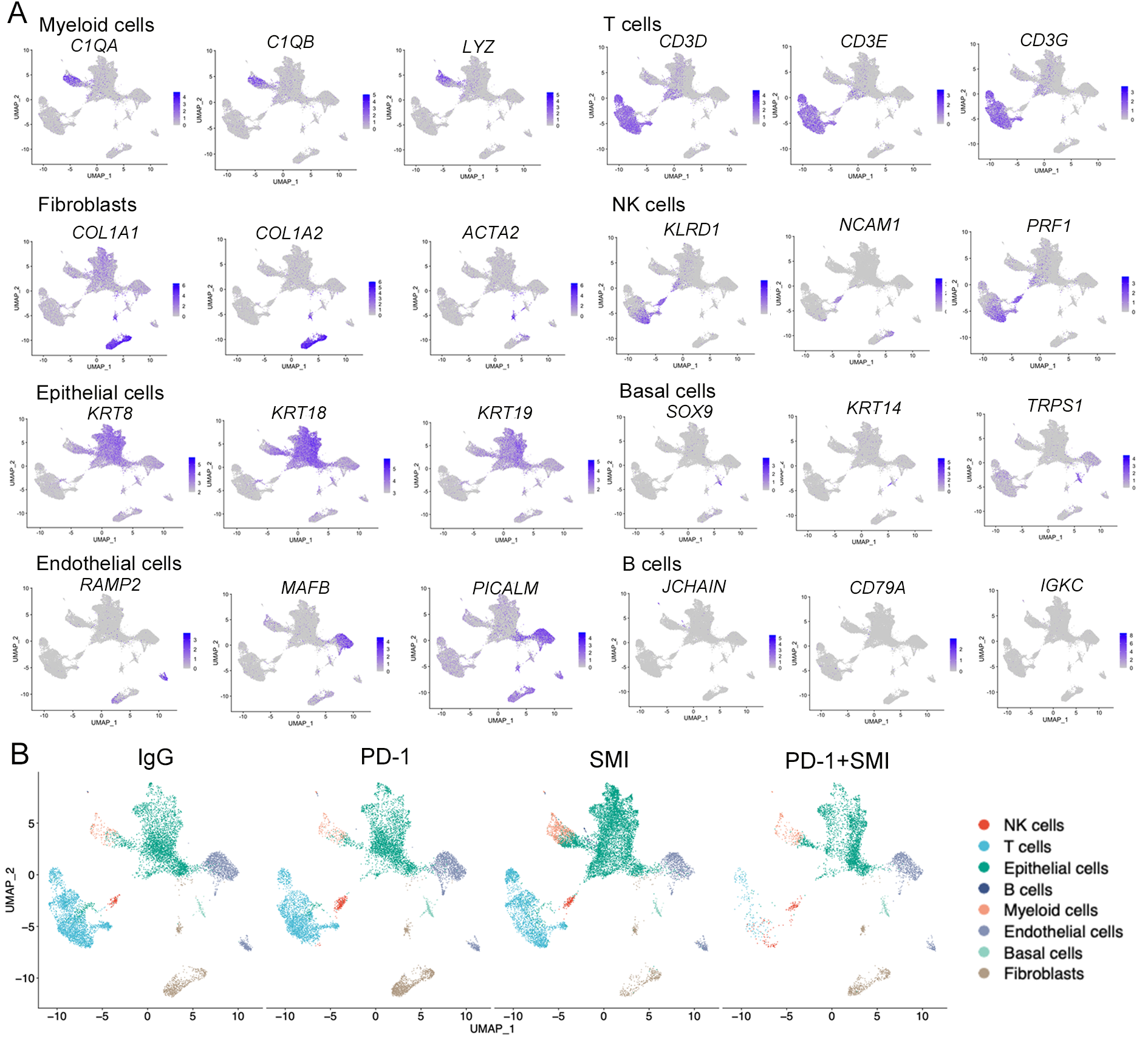


**Fig. S8** Single-cell transcriptome profiles of different treatments in the humanized mouse model. (A) The expression of marker genes for eight cell types. (B) Uniform manifold approximation and projection (UMAP) projection within each sample origin. IgG: immunoglobulin G isotype control; PD-1: PD-1 immune-checkpoint blockade antibody; SMI: SMI monotherapy; PD-1+SMI: combination of anti-PD-1 and SMI.


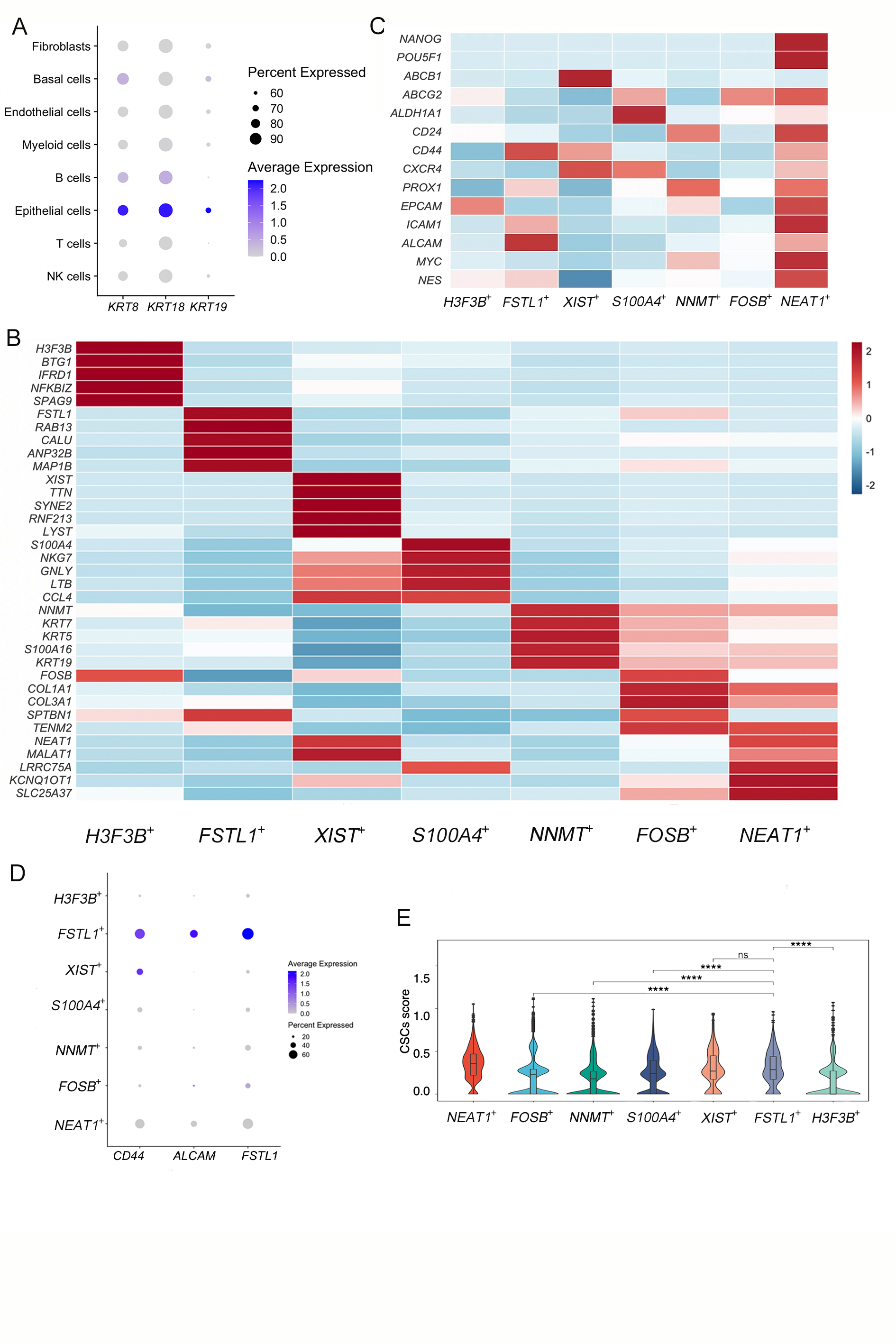


**Fig. S9** Seven subclusters of malignant epithelial cells were identified upon different treatments. (A) Dot plot showing the expression of *KRT8*, *KRT18*, and *KRT19* in eight cell types. (B) Heatmap of differentially expressed genes (DEGs) among seven malignant epithelial subclusters. *NEAT1*^+^: *NEAT1*^+^ tumor cells; *FOSB^+^*: *FOSB^+^* tumor cells; *NNMT^+^*: *NNMT^+^* tumor cells; *S100A4^+^*: *S100A4^+^* tumor cells; *XIST^+^*: *XIST^+^* tumor cells; *FSTL1^+^*: *FSTL1^+^* tumor cells; *H3F3B^+^*: *H3F3B^+^* tumor cells. (C) Heatmap showing the expression of cancer stem cells (CSCs) genes in seven malignant epithelial subclusters. (D) Dot plot showing the expression of *CD44*, *ALCAM*, and *FSTL1* in seven malignant epithelial subclusters. (E) Violin plot showing the *FSTL1*^+^ tumor cells had higher CSCs score compared with that in other subclusters except for *NEAT1*^+^ and *XIST*^+^ tumor cells.


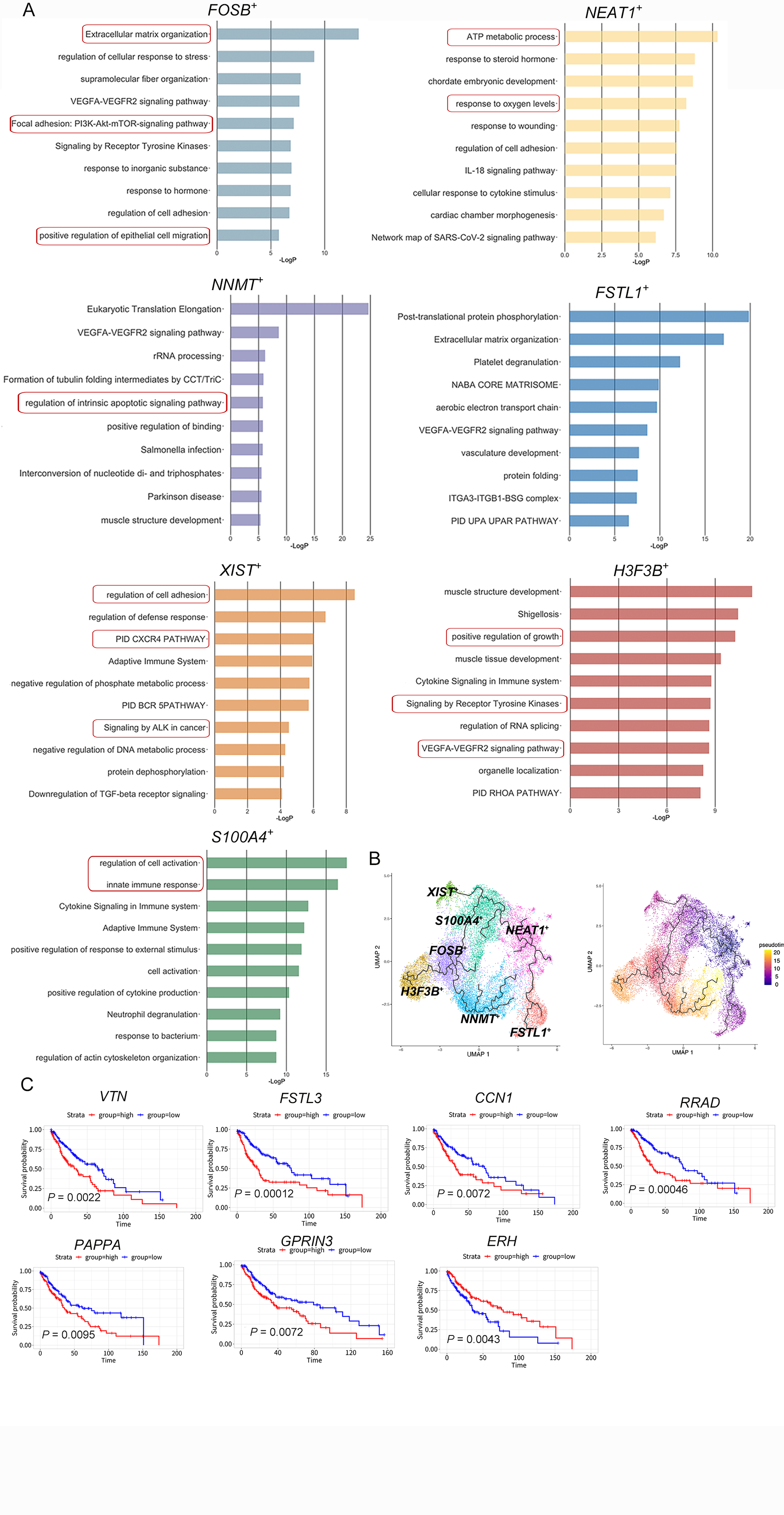


**Fig. S10** Transcriptome heterogeneous and dynamics in seven malignant epithelial subclusters. (A) Top 10 biological pathways of seven malignant epithelial clusters based on DEGs using Metascape analysis. *NEAT1*^+^: *NEAT1*^+^ tumor cells; *FOSB^+^*: *FOSB^+^* tumor cells; *NNMT^+^*: *NNMT^+^* tumor cells; *S100A4^+^*: *S100A4^+^* tumor cells; *XIST^+^*: *XIST^+^* tumor cells; *FSTL1^+^*: *FSTL1^+^* tumor cells; *H3F3B^+^*: *H3F3B^+^* tumor cells. (B) Unsupervised transcriptional trajectory of seven malignant epithelial subsets predicted by Monocle 3. (C) High level of *FOSB*^+^ tumor cell-associated genes (*FSTL3* and *VTN*), *NEAT1*^+^ tumor cell-associated genes (*PAPPA*, *CCN1*, and *RRAD*), and *XIST*^+^ tumor cell-associated genes (*GPRIN3*) predicted poor prognosis in TCGA-LUSC.htseq_counts.tsv (n = 550 samples), whereas high level of *NNMT*^+^ tumor cell-associated genes (*ERH*) exhibited higher overall survival. Log-rank *P* < 0.05 was considered as statistically significant.

**
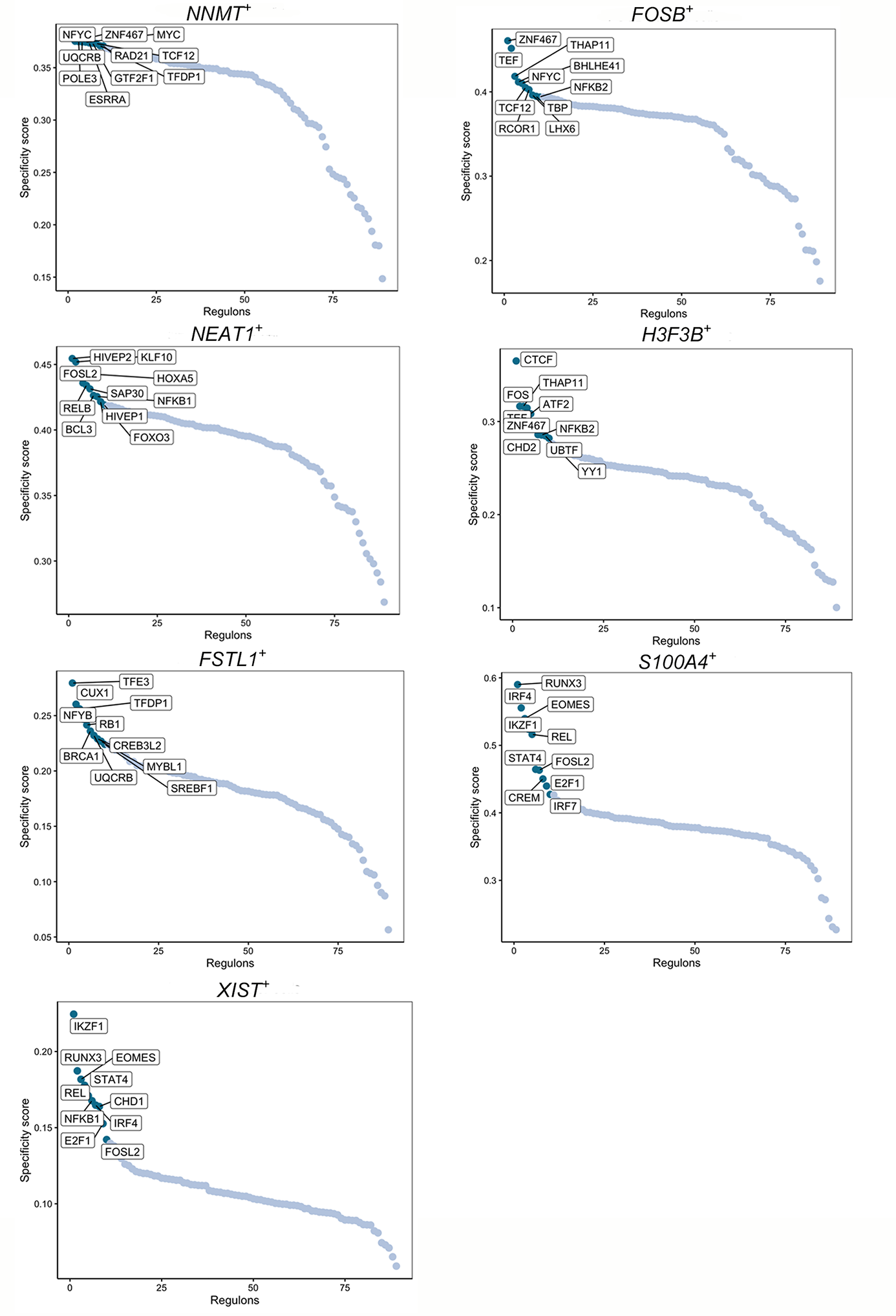
**

**Fig. S11** The top 10 Transcription factors (TFs) in each malignant epithelial subcluster were inferred using SCENIC analysis. *NEAT1*^+^: *NEAT1*^+^ tumor cells; *FOSB^+^*: *FOSB^+^* tumor cells; *NNMT^+^*: *NNMT^+^* tumor cells; *S100A4^+^*: *S100A4^+^* tumor cells; *XIST^+^*: *XIST^+^* tumor cells; *FSTL1^+^*: *FSTL1^+^* tumor cells; *H3F3B^+^*: *H3F3B^+^* tumor cells.

**
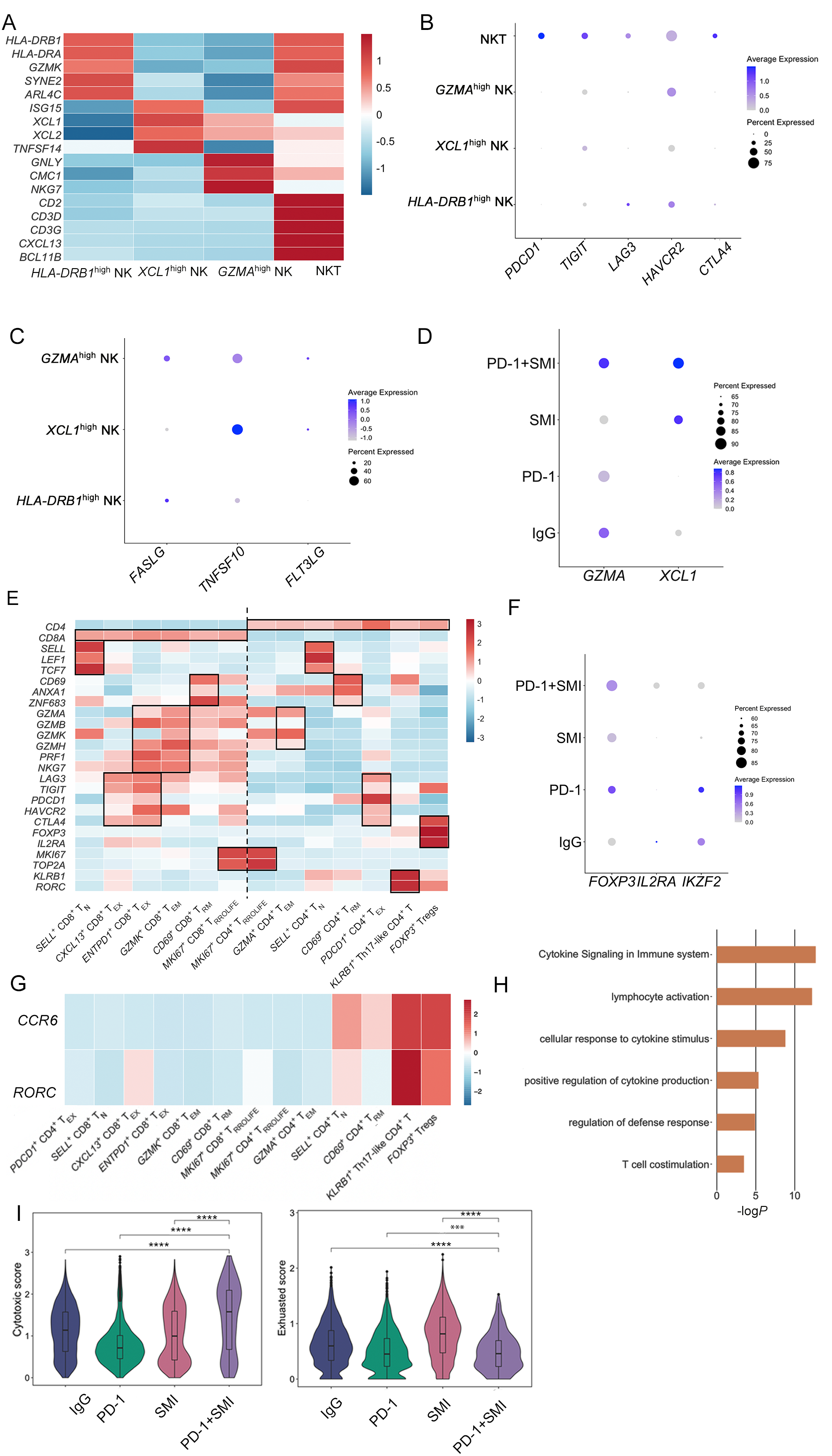
**

**Fig. S12** Single-cell transcriptome profiles of NK and T cells. (A) Heatmap of DEGs among four NK cell subclusters. (B) Dot plot showing expression of *PDCD1*, *TIGIT*, *CTLA4*, *LAG3*, and *HAVCR2* in four NK clusters. (C) Dot plot showing expression of *FASLG*, *TNFSF10*, and *FLT3LG* in *GZMA*^high^ NK, *HLA-DRB1*^high^ NK, *XCL1*^high^ NK clusters. (D) Dot plot showing expression of *GZMA* and *CMC1* in four different treatments. IgG: immunoglobulin G isotype control; PD-1: PD-1 immune-checkpoint blockade antibody; SMI: SMI monotherapy; PD-1+SMI: combination of anti-PD-1 and SMI. (E) Heatmap of T cell respective markers among thirteen T cell subclusters. (F) Dot plot showing expression of *FOXP3*, *IL2RA*, and *IKZF2* in four different treatments of Tregs. (G) Heatmap showing express of *CCR6* and *RORC* among thirteen T cell subclusters. (H) Top biological pathways of *KLRB1*^+^ CD4^+^ T helper 17-like T cells (*KLRB1*^+^ Th17-like CD4^+^ T cells) based on DEGs using Metascape analysis. (I) Violin plot showing the mean score of the cytotoxic or exhausted signature across four different treatments in T and NK cells. ^***^*P* < 0.001, ^****^*P* < 0.0001.

**
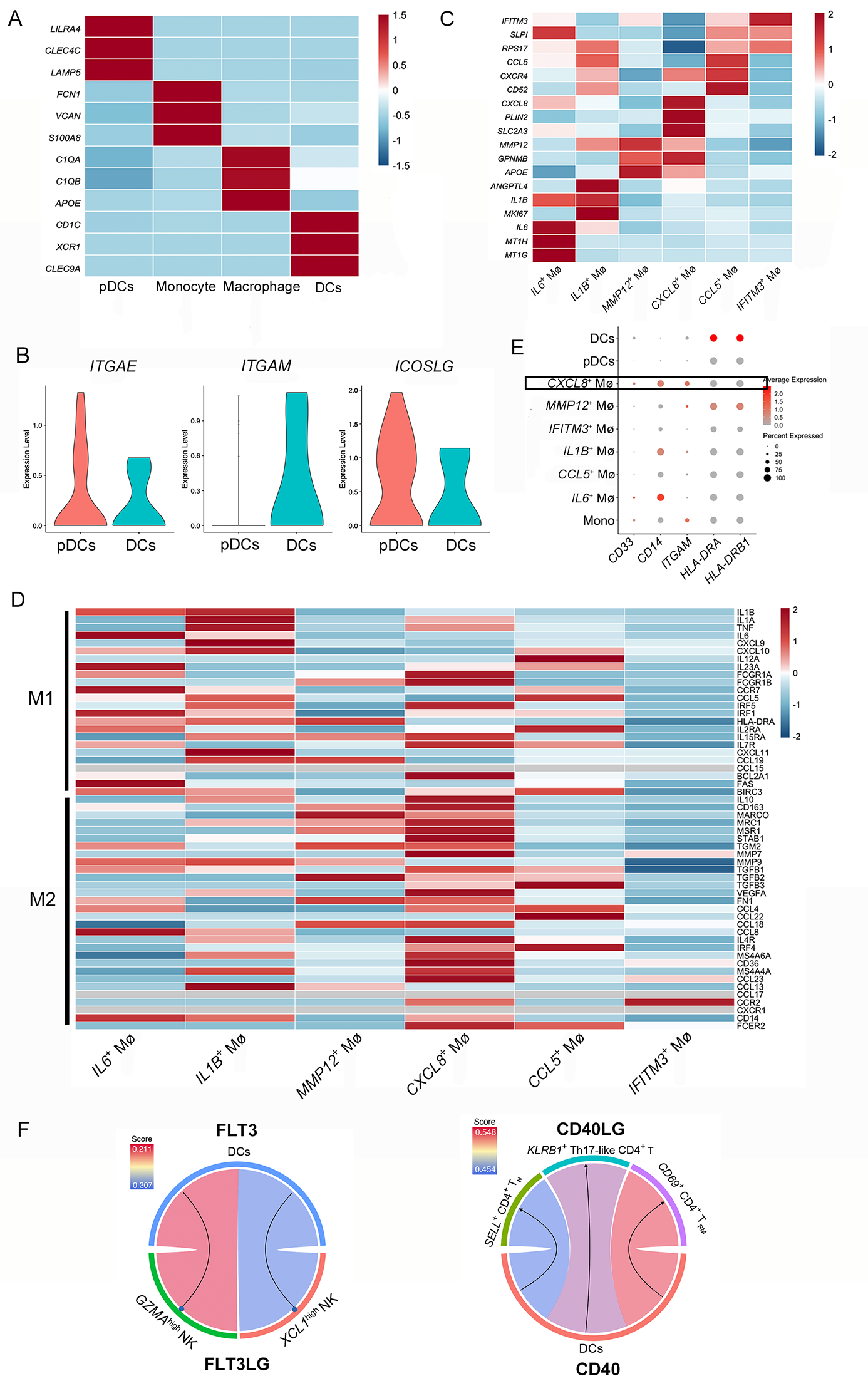
**

**Fig. S13** Single-cell transcriptome profiles of myeloid cells. (A) Heatmap of DEGs among macrophages, monocytes, classical DCs, and plasmacytoid DCs (pDCs). (B) Violin plot showing expression of *ITGAE*, *ITGAM*, and *ICOSLG* in classical DCs and pDCs. (C) Heatmap of DEGs among six macrophages subclusters. (D) Heatmap describing the expression of M1 and M2 feature genes in six macrophage subclusters. (E) Dot plot showing expression of *CD33, CD14, ITGAM, HLA-DRA,* and *HLA-DRB1* in myeloid subclusters. (F) Chord diagram showing the ligand-receptor pair FTL3-FTL3LG involved in *GZMA*^high^ NK cells and DCs and between *XCL1*^high^ NK cells and DCs, and the ligand-receptor pair CD40-CD40LG involved in DCs and *CD69*^+^ CD4^+^ T_RM_, *KLRB1*^+^ CD4^+^ Th17-like T, and *SELL*^+^ CD4^+^ T_N_ cells.

**
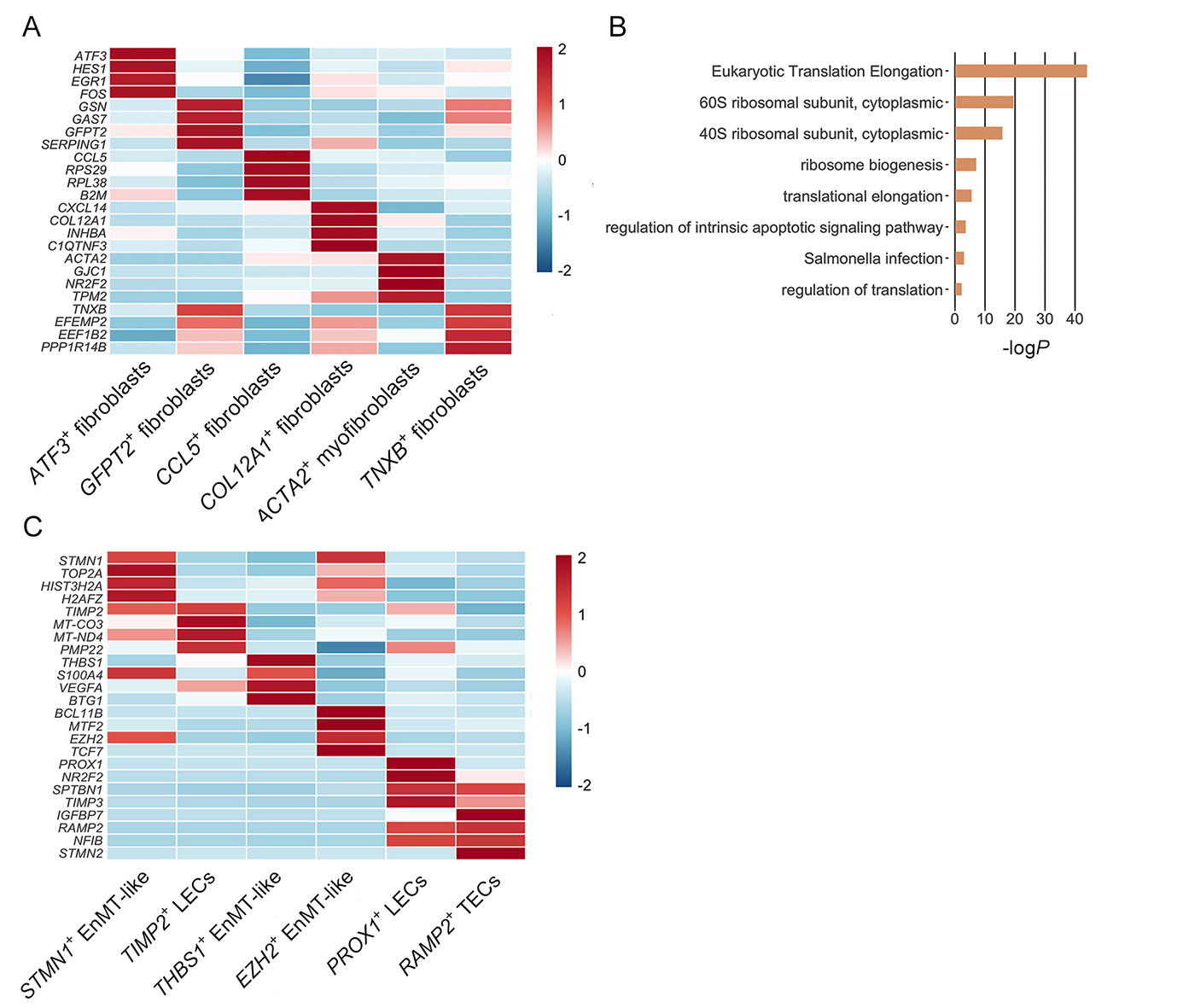
**

**Fig. S14** Single-cell transcriptome profiles of fibroblast and endothelial cells. (A) Heatmap of DEGs among fibroblast sublcusters. (B) Top biological pathways of based on DEGs of *ACTA2*^+^ myofibroblasts from the PD-1+SMI group using Metascape analysis. (C) Heatmap of DEGs among endothelial sublcusters.
