# Supplementary material for: Single-cell RNA sequencing reveals enhanced antitumor immunity after combined application of PD-1 inhibitor and Shenmai injection in non-small cell lung cancer
Source: Cell Commun Signal. 2023 Jul 10;21:169. doi: 10.1186/s12964-023-01184-3 (PMC10332015; doi:10.1186/s12964-023-01184-3)
Supplement: Supplementary file 3 — Additional file 2: Table S1 The representative marker genes of eight cell types. Table S2 Biological pathways analysis using Metascape analysis. Table S3 Top ten TFs in the tumor subclusters. Table S4 The representative marker genes of T and NK cell subclusters. [file 12964_2023_1184_MOESM2_ESM.docx]

**Additional file 2**

**Table S1** The representative marker genes of eight cell types.

| **Cluster** | **Marker gene** | **Reference** |
| --- | --- | --- |
| Epithelial cell | *KRT8*, *KRT18*, *KRT19* | PMID: 32385277, 30556162 |
| Basal cell | *SOX9*, *KRT14*, *TPRS1* | PMID: 32042191, 23776679 |
| Endothelial cell | *RAMP2*, *MAFB*, *PICALM* | PMID: 32307629, 23229650, 33144684 |
| Fibroblast | *COL1A1*, *COL1A2*, *ACTA2* | PMID: 32385277, 33953163 |
| T cell | *CD3D*, *CD3E*, *CD3G* | PMID: 32385277, 33144684 |
| B cell | *CD79A*, *JCHAIN*, *MS4A1* | PMID: 32385277, 33953163 |
| NK cell | *KLRD1*, *NCAM1*, *PRF1* | PMID: 32385277, 33144684 |
| Myeloid cell | *C1QA*, *C1QB*, *LYZ* | PMID: 33144684 |

**Table S2** Biological pathways analysis using Metascape analysis.

| **Pathway** | **Gene** |
| --- | --- |
| Extracellular matrix organization | *APP*，*CAPNS1*，*SERPINH1*，*COL1A1*，*COL3A1*，*COL4A2*，*EFEMP1*，***ITGA3***，*LOXL2*，*PLOD2*，*SPARC*，***SPP1***，*TIMP2*，***VTN***，*ADAM9*，*CDKN1A*，*IL6ST*，*DNAJB6*，*CCDC80*，*PTPRF*，*SPTBN1*，*IQGAP1*，*CRIM1*，*UGCG*，*CLIC4*，*CXCL2*，*IER3*，*LRRFIP1*，***STAT1***，*MAP1B*，***ATF4***，***HIF1A***，*RDH10* |
| Focal adhesion: PI3K-Akt-mTOR-signaling pathway | ***ATF4***，*CDKN1A*，*COL1A1*，*COL3A1*，*COL4A2*，*EIF4B*，***HIF1A***，***ITGA3***，***SPP1***，***VTN***，***CDK6***，*SGK1*，***STAT1***，*FLNA*，*MYH9*，*EFEMP1*，*TXNRD1*，*ALDH1A2*，***DNAJB6***，*RDH10*，*ADAM9*，*ANTXR1* |
| Positive regulation of epithelial cell migration | *RHOB*，*ETS1*，***HIF1A***，***ITGA3***，*SPARC*，*ADAM9*，*PLK2*，*APP*，*CD74*，*COL1A1*，***VTN***，*NT5E*，*BHLHE40*，*COL4A2*，***STAT1***，*CDKN1A*，*FOSB* |
| ATP metabolic process | *ATP1B1*，*HSPA1A*，***ATP6***，***COX1***，***COX2***，***COX3***，*CYTB*，***ND1***，***ND3***，***ND4***，***ND5***，*SLC16A3*，*DUSP1*，*TP53*，*COL1A1*，*IL6*，*DHRS3*，*ABL2*，*FUS*，*CTNNB1*，*SLC3A2*，*SLC25A37*，*ADM*，*CALR*，*CCN2* |
| Response to oxygen levels | *ADM，ATP1B1*，*ZFP36L1*，*COL1A1*，*CCN2*，*ATP6*，*ND4*，*ND5*，*PLAT*，*TP53*，*MALAT1*，*KLF2*，*HEG1*，*EIF5A*，*CALR*，*CTNNB1*，*NRP2*，*AGRN* |
| regulation of intrinsic apoptotic signaling pathway | *CAV1*，*ENO1*，*HSPB1*，*PPIA*，***RPL26***，***VDAC2***，*NUPR1*，*GSTP1*，*TNFRSF12A*，***GADD45A***，*UCHL1*，*ANXA2*，*ANXA3*，*SLPI*，*PPP1R14B*，*CKS1B*，*IL18*，*TXN*，*FHL2* |
| regulation of cell adhesion | *ATM*，*RUNX3*，*ETS1*，*ITGA4*，*PDE3B*，*PIK3R1*，*PTPRC*，***TGFB1***，*UTRN*，***CXCR4***，*SEMA4D*，*ZBTB1*，*MACF1*，*PTPN22*，*PAG1*，*AKNA*，*TSC22D3*，***TNFAIP3***，*USP15*，*PARP14*，*ZFP36L2*，*JUN*，*KMT2A*，*PRRC2C*，*BCL11B*，*CD2*，*IKZF3*，*NR3C1*，*SETX*，*HEXIM1*，*REL* |
| PID CXCR4 PATHWAY | *ITGA4*，*PIK3R1*，*PTPRC*，*RGS1*，***CXCR4***，*PAG1*，*REL*，***TNFAIP3***，*PDE3B*，*ARHGEF1*，*ADGRE5*，*PDE4D* |
| Signaling by ALK in cancer | *PIK3R1*，*RANBP2*，***EML4***，*RNF213*，*GOLGB1*，*PPP2R5C*，***TGFB1***，*AKAP9*，*SYNE2* |
| positive regulation of growth | *CDC42*，*DDX3X*，*EIF4G2*，*H3-3A*，*H3-3B*，*PAFAH1B1*，***VEGFA***，*BASP1*，*NIPBL*，*CHD7*，*NDEL1*，*H3-5*，*BTG1*，*PTEN*，*YY1*，***SPAG9***，*CITED2*，*ACTB*，*ATP2B1*，*CALM1*，*HSPA5*，*UBB*，*ZFAND5*，*BTG2*，*TIPARP*，*MSN*，*PLEK*，*ROCK1*，*PICALM*，*ARPC2*，*CYRIB*，*SKIL*，*RAB8B*，*DDX5*，*MCL1*，*PDCD10*，*IER5*，*JUN*，*ERBIN*，*FOS*，*RAB7A*，*EGR1*，*UBE2B*，*WNK1*，*CPEB2* |
| Signaling by Receptor Tyrosine Kinases | *ACTB*，*CALM1*，***CDC42***，*CHD*，*CLTC*，*EGR1*，*FOS*，*HNRNPF*，*RAP1B*，*ROCK1*，*UBB*，***VEGFA***，*PSENEN*，*ERBIN*，*HSPA5*，*BASP1*，*MSN*，*PTEN*，*QKI*，*RAB14* |
| VEGFA-VEGFR2 signaling pathway | ***CDC42***，*CLTC*，*EGR1*，*EIF4G2*，*JUN*，*NFKBIA*，*RAP1B*，*ROCK1*，***VEGFA***，*QKI*，*RBM39*，*UBAP2L*，*IER5*，*CALM1*，*MCL1*，*BTG2*，*ACTB*，*UBB*，*FOS*，*CHMP4B* |
| Cytokine signaling in Immune system | ***TNFSF8***，*IFI6*，*IFI27*，*IFIT1*，*IFIT3*，*JUNB*，*LIF*，*LTB*，*MX1*，*EIF2AK2*，*RORA*，*CCL20*，*IRS2*，***TNFRSF25***，***TNFSF14***，*ISG15*，*TRIM22*，***TNFSF13B***，*USP18*，*RSAD2*，*CXCR4*，*IFI44*，*IVNS1ABP*，*IFI44L*，*ZFP36*，*RBPJ*，*THBS1* |
| T cell costimulation | ***TNFSF14***，***TNFSF13B***，*KLRK1* |

**Table S3** Top ten TFs in the tumor subclusters.

| **Tumor subcluster name** | **TFs** |
| --- | --- |
| *FOSB*^+^ tumor cells | ZNF467, TEF, THAP11, NFYC, BHLHE41, TCF12, RCOR1, LHX6, TBP, NFKB2 |
| *NEAT1*^+^ tumor cells | HIVEP2, KLF10, HOXA5, FOSL2, RELB, NFKB1, BCL3, SAP3, HIVEP1, FOXO3 |
| *NNMT*^+^ tumor cells | NFYC, ZNF467, MYC, POLE3, UQCRB, ESRRA, GRF2F1, TCF12, RAD21, TFDP1 |
| *FSTL1*^+^ tumor cells | TFE3, CUX1, TFDP1, NFYB, RB1, BRCA1, UQCRB, CREB3L2, MYBL1, SREBF1 |
| *XIST*^+^ tumor cells | IKZF1, RUNX3, EOMES, STAT4, REL, NFKB1, CHD1, IRF4, E2F1, FOSL2 |
| *H3F3B*^+^ tumor cells | CTCF, FOS, THAP11, TEF, ATF2, ZNF467, NFKB2, CHD2, UBTF, YY1 |
| *S100A4*^+^ tumor cells | RUNX3, IRF4, EOMES, IKZF1, REL, STAT4, FOSL2, CREM, E2F1, IRF7 |

**Table S4** The representative marker genes of T and NK cell subclusters.

| **T/NK cell cluster names** | **Representative genes** |
| --- | --- |
| *SELL*^+^ CD8^+^ TN | *CD8, SELL, CCR7, TCF7, IL7R* |
| *CXCL13*^+^ CD8^+^ TEX | *CD8, CXCL13, TNFRSF9, NMB, TNFRSF18* |
| *ENTPD1*^+^ CD8^+^ TEX | *CD8, ENTPD1, LAYN, KLRC1, HOPX* |
| *GZMK*^+^ CD8^+^ TEM | *CD8, GZMK, LIME1, COTL1, MCTP2* |
| *CD69*^+^ CD8^+^ TRM | *CD8, CD69, IFIT2, PIK3R1, ZNF683* |
| *MKI67*^+^ CD8^+^ TPROLIFE | *CD8, MKI67, TOP2Q, STMN1, ASPM* |
| *MKI67*^+^ CD4^+^ TPROLIFE | *CD4, MKI67, TOP2A, STMN1, ASPM* |
| *GZMA*^+^ CD4^+^ TEM | *CD4, GZMA, GZMK, GZMM, KLRG1* |
| *SELL*^+^ CD4^+^ TN | *CD4, CCR7, SELL, IL7R, KLF2, KLF3* |
| *CD69*^+^ CD4^+^ TRM | *CD4, CD69, IL7R, ZFP36L2, GPR183* |
| *PDCD1*^+^ CD4^+^ TEX | *CD4, PDCD1, LAG3, HAVCR2, HOPX* |
| *FOXP3*^+^ Tregs | *CD4, FOXP3, IL2RA, LTB, IFI27* |
| *KLRB1*^+^ Th17-like CD4^+^ T cells | *CD4, KLRB1, CCL20, CTSL, RORC* |
